# Oxypeucedanin: Chemotaxonomy, Isolation, and Bioactivities

**DOI:** 10.3390/plants10081577

**Published:** 2021-07-30

**Authors:** Javad Mottaghipisheh

**Affiliations:** Center for Molecular Biosciences (CMBI), Institute of Pharmacy/Pharmacognosy, University of Innsbruck, Innrain 80-82, 6020 Innsbruck, Austria; Javad.Mottaghipisheh@uibk.ac.at

**Keywords:** furanocoumarins, oxypeucedanin, chemotaxonomy, isolation, bioactivities, pharmacokinetics

## Abstract

The present review comprehensively gathered phytochemical, bioactivity, and pharmacokinetic reports on a linear furanocoumarin, namely oxypeucedanin. Oxypeucedanin (OP), which structurally contains an epoxide ring, has been majorly isolated from ethyl acetate-soluble partitions of several genera, particularly *Angelica*, *Ferulago*, and *Prangos* of the Apiaceae family; and *Citrus,* belonging to the Rutaceae family. The methanolic extract of *Angelica dahurica* roots has been analytically characterized as the richest natural OP source. This naturally occurring secondary metabolite has been described to possess potent antiproliferative, cytotoxic, anti-influenza, and antiallergic activities, as assessed in preclinical studies. In order to explore potential drug candidates, oxypeucedanin, its derivatives, and semi-synthetically optimized analogues can be considered for the complementary assessments of biological assays.

## 1. Introduction

Coumarins, as a broad class of secondary metabolites, are divided into diverse derivatives according to their structural categories, such as simple coumarins, 4-phenylcoumarins, pyranocoumarins, benzocoumarins, and furanocoumarins (syn. furocoumarins) [1]. The fundamental furanocoumarin structure is based on fusing a furan ring to umbelliferone (7-hydroxycoumarin). According to the furan ring position, linear and angular derivatives are generated through two biosynthesis pathways, including phenylpropanoid and mevalonic acid [1].

Aside from several documented biological properties, furanocoumarins are mostly distinguished for photosensitizing potencies. Some of the linear furanocoumarins, specifically psoralen, bergapten, and xanthotoxin, are phototoxic [2]. Phototoxicity of these naturally occurring compounds can be employed beneficially to treat some dermatitis disorders. Psoralen, the simplest linear furanocoumarin, is used to remedy inflammatory skin diseases such as psoriasis. This constituent induces inflammatory cell apoptosis via a UVA interaction, suppressing DNA synthesis, and cell proliferation [3,4]. Notwithstanding this activity, furanocoumarins are rarely allergenic; among them, isobergapten (linear) and sphondin (angular) are verified as the major allergenic furanocoumarins [2].

**Figure 1:**
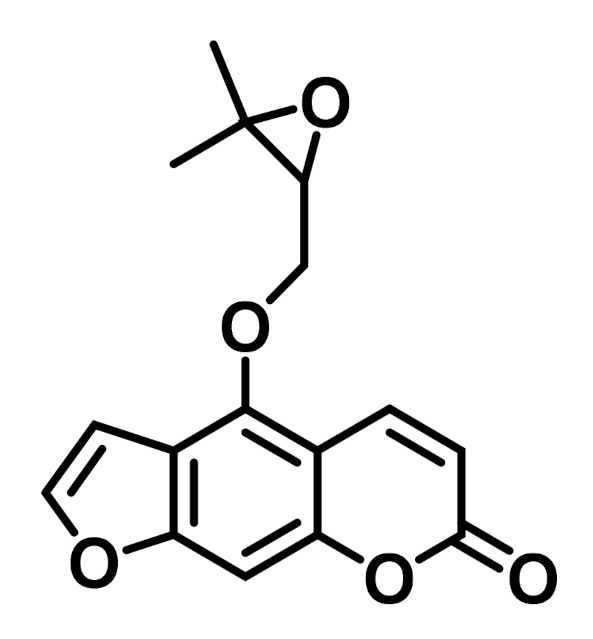
The chemical structure of oxypeucedanin.

Most of the furanocoumarins have been chemo-taxonomically characterized from the Apiaceae, Rutaceae, and Fabaceae families [5,6]. Oxypeucedanin (OP, C_16_H_14_O_5_, molecular weight: 286.28 g/mol, 4-[(3,3-dimethyloxiran-2-yl)methoxy]furo[3,2-g]chromen-7-one) is a linear furanocoumarin containing an epoxide ring in which, at position 5, 7H-furo[3,2-g][1]benzopyran-7-one is substituted by a [(2S)-3,3-dimethyloxiran-2-yl]methoxy group (Figure 1) [7].

Considering the substantial furanocoumarin contents comprising OP in plant foods [1,8,9,10], and the diverse biological effects on human health, investigations of phytochemicals and their bioactivities are crucial approaches. The present context aims at overviewing all the phytochemical (preparative and analytical) and pharmacological (including pharmacokinetic) studies of OP that have been carried out. “Oxypeucedanin” was applied as a keyword to seek the correlated data through the English-language papers indexed in the Web of Science, SciFinder, and PubMed scientific databases, in which all the plants’ parts were considered (accessed date: 25 May 2021).

## 2. Phytochemical Studies of Oxypeucedanin

Several experiments have previously been performed to isolate, purify, and identify OP from various plant species entirely belonging to the Apiaceae and Rutaceae families.

Some of the plants possessing OP are being consumed as foods and spices; for instance, the leaves of dill (*Anethum graveolens*), *Ferulago* spp., *Angelica archangelica*, and *Prangos* spp. (Apiaceae), along with the renowned *Citrus* fruits (Rutaceae). Traditional applications of the major plants containing OP will be mentioned in the following sections, but for example, the consumption of *Angelica dahurica* roots as a toothache reliever and for the treatment of the common cold in China, and the use of *Prangos* leaves as tonic, carminative, and anthelmintic agents in Iran can be mentioned.

High-performance liquid chromatography coupled with both ultraviolet (HPLC-UV) and diode array detectors (HPLC-DAD) have been utilized for analytical assessment of OP from plants, during which the qualification and quantification analysis were mostly accomplished on *Angelica dahurica* roots (Apiaceae). The following sections present the phytochemical aspects of OP, with detailed information documented in Table 1 and Table 2.

### 2.1. Preparative Analysis of Oxypeucedanin

#### 2.1.1. Isolation and Purification of Oxypeucedanin from the Apiaceae Family

The Apiaceae (syn. Umbelliferae) family has been identified as one of the major natural sources of coumarin derivatives [11]. So far, OP has been isolated from 23 species belonging to 11 different genera by utilizing diverse separation techniques. Among them, the genera of *Angelica*, *Ferulago*, and *Prangos* have been investigated in six, five, and three phytochemical studies, respectively, leading to isolation of OP. Overall, 50% of the identified OP has been isolated from root parts of the plants, while the best soluble partitions were ethyl acetate (EtOAc, 12 cases), chloroform (CHCl_3_, 7 cases), and dichloromethane (CH_2_Cl_2_, 6 cases). Table 1 documents all the preparative information on OP purified from different plant species.

*Anethum graveolens*

Oxypeucedanin has been separated from whole part of *A. graveolens* and its *n*-hexane (NHEX)-soluble fraction by applying vacuum liquid chromatography (VLC) and reverse-phase (RP) preparative thin-layer chromatography (PTLC) [12].

*Angelica* spp.

Medium-pressure liquid chromatography (MPLC) on silica gel has previously been exploited to isolate OP from the root CHCl_3_-soluble fraction of *A. archangelica* [13]. In traditional Chinese medicine, the roots of *A. dahurica* are renowned as “Hangbaizhi” or “Baizhi”, and famed for their therapeutic properties, specifically treatment of the common cold and toothaches, and as a food spice [14]. The root part of this species has been described to contain several furanocoumarins; however, among them, the presence of OP was proven by many studies compared to the other natural sources, thus it can be introduced as the major OP origin [15].

Column chromatography on silica gel (CC) has been used for the isolation of OP from the root methanolic fraction of *A. dahurica* [16,17,18]. This compound has been isolated from ethanolic and *n*-butanol (BuOH)-soluble partitions of this species by exploiting CC [19] and recrystallization [20], respectively; as well as from the CHCl_3_ and CH_2_Cl_2_ soluble fractions using CC [21] and recrystallization methods [22], and CC on silica gel [23,24], respectively.

Sephadex^®^ LH-20 (SLH) has played a key role in isolation and purification of diverse classes of natural products [25]. This method has further been used for the isolation and separation of coumarins, including OP. Ethyl acetate-soluble extract of *A. dahurica* root can be considered as the richest OP source. The eluting solvent system comprising NHEX–EtOAc (3:2) through CC on silica gel [26,27], as well as SLH [28] and recrystallization [29], have been applied as the final separation procedures.

High-speed counter-current chromatography (HSCCC) is a liquid stationary-phase chromatographic technique in which the mixtures are separated according to their polarity into two immiscible solvent systems [30]. It also has been utilized as an OP purification instrument, while a two-phase solvent system of NHEX–EtOAc–MeOH–H_2_O was optimized and employed [31,32,33]. OP also was isolated from nonpolar soluble partitions of *A. dahurica* using semipreparative HPLC [34] and octadecyl silica gel column chromatography (ODS-CC) [35].

Numerous chromatographic steps were applied to isolate OP from EtOAc extract of *A. furcijuga* flower, whilst HPLC was chosen as the final stage [36]. OP has been isolated from the root EtOAc fraction of *A. koreana* [37], as well as from the methanolic and CH_2_Cl_2_ soluble partitions of *A. pancicii* by utilizing flash chromatography (FC) and HPLC [38]. The fruit part of *A. purpurascens* has been extracted via various organic solvents, and the NHEX fraction was subsequently subjected to CC on silica gel, leading to OP purification [39].

*Diplolophium buchanani*

Dichloromethane partition of *D. buchanani* leaf has previously been chromatographed via a final recrystallization method; however, in this study, centrifugal partition chromatography was primarily used [40].

*Ducrosia anethifolia*

In a phytochemical study, OP was isolated and characterized from the CHCl_3_-soluble fraction of the aerial part of *D. anethifolia* by means of CC on silica gel, MPLC, and centrifugal preparative thin-layer chromatography (CPTLC) [41].

*Levisticum officinale*

The root parts of *L. officinale* have recently been extracted using different solvents. After solvent–solvent partitioning, the EtOAc fraction was subjected to CC via NHEX–EtOAc (100:0 to 0:100) and EtOAc–MeOH (100:0 to 80:20) as mobile phase systems, and OP was subsequently isolated [42].

*Ferulago* spp.

Various nonvolatile and volatile components of *F. angulata* (syn. *F. trifida*) have been identified as its significant and predominant constituents [43]. The root part was fractionated, the NHEX-soluble partition was chromatographed, and OP was accordingly isolated by preparative thin-layer chromatography (PTLC) [44]. The CHCl_3_-partition of the root was also separated by CC on silica gel, and OP was purified [45,46]. Oxypeucedanin has been isolated and identified from the NHEX aerial part of *F. bernardii*, whilst CC on silica gel (mobile phase: PET–EtOAc–MeOH), and lastly recrystallization (from H_2_O–EtOH), were exploited [47].

The aerial and root parts of *F. capillaris* have been further subjected to separation of their constituents. Oxypeucedanin was isolated from the NHEX-soluble partition, considering that CC on silica gel with a gradient solvent system including NHEX–EtOAc was applied [48]. OP has further been separated from the PET soluble fraction of the *F. humulis* rhizome. In this recent study, CC (mobile phase: NHEX‒EtOAc; EtOAc‒MeOH) and PTLC (elution system: cyclohexane‒EtOAc 2:1) were utilized as chromatographic tools [49]. The separation procedure, including CC on silica gel through eluting solvent systems of NHEX–CHCl_3_ and CHCl_3_–EtOAc, and recrystallization, led to the isolation of OP from the root EtOAc fraction of *F. subvelutina* [50].

*Harbouria trachypleura*

Oxypeucedanin has been isolated from the methanolic fraction of the aerial part of *H. trachypleura* by applying the following separation techniques: vacuum liquid chromatography (VLC, mobile phase: NHEX–EtOAc), FC (mobile phase: CH_2_Cl_2_–Me_2_CO 97:3), CC (mobile phase: CH_2_Cl_2_–Me_2_CO 97:3 to 4:1), RP-VLC (mobile phase: H_2_O–MeOH 50:50 to 100:0), and PTLC (mobile phase: CH_2_Cl_2_–Me_2_CO 97:3) [51].

*Ostericum koreanum*

The dried root of *O. koreanum* is known as “Osterici radix” in China. It has traditionally been consumed as a natural analgesic agent in oriental medicine [52]. The root parts of *O. koreanum* has been subjected for the preparative phytochemical investigations. From the CHCl_3_ and EtOAc-soluble fractions, OP was isolated and identified by using CC on Silica gel, eluting with benzene (Bz)–EtOAc (9:1) [52] and NHEX–EtOAc [53,54], respectively.

*Petroselinurn crispurn*

Four studies described isolation of OP from the flake and leaf parts of *P. crispurn*. The diethyl ether (DEE)-soluble partition of this species has been separated *via* HPLC [55,56], in addition the implementation of CC on Silica gel, SLH, and PTLC on the leaves’ CH_2_Cl_2_ [57] and EtOAc-partitions was led to purify OP [58].

*Peucedanum* spp.

The plants of the *Peucedanum* genus have been recognized for using in folk medicine to treat joint pain, sore throat, epilepsy, gastrointestinal and respiratory disorders [59]. Counter-current chromatography (CCC) has previously been used to isolate OP from the CH_2_Cl_2_-partition of *P. cervaria* fruit [59]. A gradient solvent system comprising H_2_O – MeOH was applied for the isolation of OP through semipreparative-HPLC from the root EtOAc-partition of *P. ostruthium* [60].

*Prangos* spp.

In addition to various phytochemical reports on *P. ferulacea* [11,61], Shokoohinia et al. [62] also isolated OP from the root’s Me_2_CO-extract by utilizing VLC and MPLC. Operation of CC was led to the OP isolation from the CHCl_3_-soluble partition of *P. pabularia* root [63] and the *P. uloptera* NHEX leaf fraction [64].

#### 2.1.2. Isolation and Purification of Oxypeucedanin from the Rutaceae Family

Oxypeucedanin has been isolated and characterized from six species belonging to Rutaceae family; among them, the peel’s essential oils of various *Citrus* species have been documented as the major source of this furanocoumarin (Table 1).

*Citrus* spp.

Previously, oxypeucedanin was isolated and identified from the EtOAc extract of *C. hystrix* fruit by applying first ODS-CC, then PTLC [65]. The essential oils extracted from the *C. limon* peel has been stated to contain OP. In a study performed by Arimoto et al. [66], using of CC on Silica gel and HPLC, and in the research of Dugo et al. [67] CC on Silica gel and recrystallization were led to isolation and purification of this furanocoumarin; additionally, HSCCC method has been exploited to separate OP from the Key’s (*C. aurantifolia*) and Persian lemon’s (*C. latifolia*) volatile oils [68].

*Skimmia japonica*

Preparative-TLC, and CC on Silica gel (mobile phase: NHEX – CHCl_3_ – EtOAc 8:1:1, 6:3:1) have previously been applied in the isolation of OP from the leaf’s EtOAc fraction of *S. japonica* [69].

*Zanthoxylum flavum*

In another study, an isocratic solvent system using H_2_O–MeOH (6:4) was employed through an RP-solid-phase extraction (SPE) method, in which OP was isolated and characterized as a pure compound from the root methanolic extract of *Z. flavum* [70].

**Table 1:** Isolated oxypeucedanin from different plant species.

Plant Family	Plant Species	Subjected Plant Part/Soluble Extract	Method of Isolation/Purification	Reference
Apiaceae	*Anethum graveolens*	WP/NHEX	VLC [NHEX‒EtOAc 100:0 to 90:10], VLC [NHEX–EtOAc 50:50], RP-PTLC [NHEX–EtOAc 60:40]	[12]
	*Angelica archangelica*	R/CHCl_3_	MPLC [butanone‒CHCl_3_‒DEE‒NHEX 6.9:1:1.4;90.7% to 48:6.8:10:35.2%], MPLC [THF–Propanol–MeCN–H_2_O 7.5:1.3:1.7:89.5 to 45.1:7.5:10:37.4]	[13]
	*Angelica dahurica*	R/MeOH	CC [NHEX–EtOH–MeOH], CC [NHEX–EtOH], CC[NHEX–EtOH– MeOH 5:1:1], CC [NHEX–EtOH 1:1]	[16]
		R/MeOH	CC [NHEX–EtOAc–MeOH], CC [NHEX–EtOAc–MeOH 5:1:1], CC [NHEX–EtOAc 1:1]	[17]
		R/MeOH	CC [NHEX–EtOAc 5:1, 1:1, 0:1], CC [NHEX–EtOAc 5:1]	[18]
		R/EtOH	CC [NHEX–Me_2_CO 20:1, 15:1, 10:1, 7:1, 4:1, 2:1, 1:1; CHCl_3_–MeOH 10:1, 8:1, 6:1, 4:1, 2:1, 1:1], CC [NHEX–Me_2_CO 20:1, 15:1, 10:1, 8:1, 6:1, 4:1, 2:1], CC [NHEX–Me_2_CO 14:1, 4:1]	[19]
		R/*n*-BuOH	CC [NHEX‒Me_2_CO 20:1 to 2:1], RP-CC [MeOH‒H_2_O 75:25], RP-HPLC [MeCN‒H_2_O 40:60], recrys.	[20]
		R/CHCl_3_	CC	[21]
		R/CHCl_3_	CC [CHCl_3_‒Me_2_CO 1:0, 0:1], CC [NHEX-EtOAc], recrys. [DEE‒CH_2_Cl_2_]	[22]
		R/CH_2_Cl_2_	CC [NHEX–EtOAc 5:1 to 0:1]	[23]
		R/CH_2_Cl_2_	CC [NHEX–EtOAc], CC [NHEX–EtOAc 3:1]	[24]
		R/EtOAc	CC [NHEX–EtOAc 3:2]	[26,27]
		R/EtOAc	CC [CH_2_Cl_2_‒MeOH 100:0 to 0:100], ODS-CC [MeOH‒H_2_O 50:50 to 95:5], SLH [MeOH]	[28]
		R/EtOAc	HSCCC [NHEX–EtOAc–MeOH–H_2_O 5:5:4:6]	[31]
		R/EtOAc	HSCCC [NHEX–EtOAc–MeOH–H_2_O 1:1:1:1, 5:5:4.5:5.5]	[32]
		R/EtOAc	HSCCC [NHEX–EtOAc–MeOH–H_2_O 1:1:1:1, 5:5:4:6]	[33]
		R/EtOAc	CC [NHEX–EtOAc 20:80 to 0:100], CC [EtOAc–MeOH 100:0 to 0:100], recrys.	[29]
		R/PET	CCC [NHEX–MeOAc–MeCN–H_2_O 4:3:4:4], HPLC	[34]
		R/NHEX	CC [NHEX–EtOAc], ODS-CC [MeOH–H_2_O 80:20]	[35]
		R/nd	nd	[15]
	*Angelica furcijuga*	Fl/EtOAc	CC [NHEX–EtOAc 10:1 to 5:1 to 1:1, to MeOH 100%], RP-CC [MeOH–H_2_O 70:30 to 80:20 to 90:10 to MeOH 100%], RP-CC [MeOH–H_2_O 60:40 to 75:25, 0:100], HPLC [MeOH–H_2_O 75:25]	[36]
	*Angelica koreana*	R/EtOAc	HPLC [MeCN–H_2_O 30:70]	[37]
	*Angelica pancicii*	R/MeOH R/CH_2_Cl_2_	FC [petrol; DEE], HPLC [MeCN–H_2_O (HCO_2_H 2%) 50:50, 65:35] HPLC [MeCN–H_2_O (HCO_2_H 2%) 50:50, 65:35]	[38]
	*Angelica purpurascens*	Fr/NHEX	CC [NHEX–EtOAc 100:0 to 0:100], CC [NHEX–EtOAc 80:20]	[39]
	*Diplolophium buchanani*	L/CH_2_Cl_2_	CPC [NHEX–EtOAc–MeOH–H_2_O 10:5:5:1], recrys. [NHEX–EtOAc]	[40]
	*Ducrosia anethifolia*	AP/CHCl_3_	CC [CHCl_3_–MeOH 100:0 to 20:80], MPLC [NHEX–CH_2_Cl_2_ 50:50 to 0:100, CH_2_Cl_2_–MeOH 100:0 to 100:0], MPLC [NHEX–EtOAc 95:5 to 0:100], CPTLC [NHEX–EtOAc 95:5 to 0:100]	[41]
	*Ferulago angulate* (syn. *F. trifida*)	R/NHEX	PTLC [CHCl_3_–Me_2_CO 95:5]	[44]
		R/CHCl_3_	CC [CHCl_3_‒EtOAc 10:0, 5:5], CC [CHCl_3_‒EtOAc 9:1]	[45,46]
	*Ferulogo bernardii*	AP/NHEX	CC [PET‒EtOAc‒MeOH 60:40], recrys. [EtOH‒H_2_O]	[47]
	*Ferulago capillaris*	AP, R/NHEX	CC [NHEX–EtOAc]	[48]
	*Ferulago humulis*	Rhizome/PET	CC [NHEX‒EtOAc; EtOAc‒MeOH], PTLC [cyclohexane‒EtOAc 2:1]	[42]
	*Ferulago subvelutina*	R/EtOAc	CC [NHEX‒CHCl_3_, CHCl_3_, CHCl_3_‒EtOAc], CC [CHCl_3_‒EtOAc 9:1, EtOAc], recrys.	[49]
	*Harbouria trachypleura*	AP/MeOH	VLC [NHEX‒EtOAc], FC [CH_2_Cl_2_–Me_2_CO 97:3], CC [CH_2_Cl_2_–Me_2_CO 97:3 to 4:1], RP-VLC [MeOH‒H_2_O 50:50 to 100:0], PTLC [CH_2_Cl_2_–Me_2_CO 97:3]	[50]
	*Levisticum officinale*	R/EtOAc	CC [NHEX–EtOAc 100:0 to 0:100; EtOAc–MeOH 100:0 to 80:20]	[42]
	*Ostericum koreanum*	R/CHCl_3_	CC [Bz‒EtOAc 9:1]	[51]
		R/EtOAc	CC [NHEX–EtOAc]	[52,53]
	*Petroselinurn crispurn*	flake/DEE	RP-CC [MeCN–H_2_O 3:2], HPLC [MeCN–H_2_O 37:63], HPLC [CHCl_3_‒MeOH 99.9:0.1]	[54]
		L/DEE	CC [NHEX–DEE 1:1], HPLC [MeCN–H_2_O 35:65]	[55]
		L/EtOAc	nd	[57]
		L/CH_2_Cl_2_	CC [PET–EtOAc 1:0, 9:1, 8:2], CC [CH_2_Cl_2_–EtOAc 9:1], CC [CH_2_Cl_2_–MeOH 9:1, 8:2, 0:1], SLH [PE–CH_2_Cl_2_–MeOH 3:2:1], SLH [PET–CH_2_Cl_2_–MeOH 4:2:1], PTLC [PET–EtOAc 7:3]	[56]
	*Peucedanum cervaria*	Fr/CH_2_Cl_2_	CCC [heptane–EtOAc–MeOH–H_2_O 3:2:3:2]	[58]
	*Peucedanum ostruthium*	R/EtOAc	HPLC [MeOH–H_2_O (HOAc 0.1%) 0:100 to 100:0]	[59]
	*Prangos ferulacea*	R/Me_2_CO	VLC [heptane–EtOAc 10:0 to 0:10], MPLC [heptane–EtOAc 7:3 to 5:5]	[61]
	*Prangos pabularia*	R/CHCl_3_	CC [NHEX–EtOAc 20:1; 10:1 to 0:1; EtOAc–MeOH 15:1 to 2:1], CC [NHEX–EtOAc 15:1]	[62]
	*Prangos uloptera*	L/NHEX	CC [NHEX–EtOAc 100:0, 1:99, 5:95, 10:99, 20:80, 40:60, 60:40, 80:20, 100:0, MeOH 100], CC [NHEX–EtOAc 20:80, 0:100, MeOH 100%], PTLC [Me_2_CO–CHCl_3_ 5:95]	[63]
Rutaceae	*Citrus hystrix*	Fr/EtOAc	ODS-CC [MeOH–H_2_O 50:50], PTLC [EtOAc–NHEX 4:1]	[64]
	*Citrus limon*	peel/EO	PTLC [EtOAc–Me_2_CO 95:5], HPLC	[65]
		peel/EO	CC [PET–EtOAc 80:20], recrys.	[66]
	*Citrus aurantifolia* & *C. latifolia*	nd/EO	HSCCC [NHEX–EtOAc–MeOH–H_2_O 6:4:5:5]	[67]
	*Skimmia japonica*	L/EtOAc	PTLC [CHCl_3_–EtOAc 8:2], PTLC [NHEX–CHCl_3_–EtOAc 7:2:1], CC [NHEX–CHCl_3_–EtOAc 8:1:1, 7:2:1, 6:3:1]	[68]
	*Zanthoxylum flavum*	R/MeOH	RP-SPE [MeOH–H_2_O 6:4]	[69]

AP: aerial part, Bz: benzene, CC: column chromatography on silica gel, CCC: counter-current chromatography, CH_2_Cl_2_: dichloromethane, CHCl_3_: chloroform, CPC: centrifugal partition chromatography, CPTLC: centrifugal preparative thin layer chromatography, EO: essential oil, EtOAc: ethyl acetate, EtOH: ethanol, DEE: diethyl ether, Fl: flower, Fr: fruit, H_2_O: water, HCO_2_H: formic acid, HPLC: high-performance liquid chromatography, HSCCC: high-speed counter-current chromatography, L: leaf, Me_2_CO: Me2CO, MeCN: acetonitrile, MeOAc: methyl acetate, MeOH: methanol, MPLC: medium pressure liquid chromatography, *n*-BuOH: *n*-butanol, NHEX: *n*-hexane; nd: not determined, ODS: octadecyl silica gel, PET: petroleum ether, PTLC: preparative thin layer chromatography, R: root, recrys.: recrystallization, RP: reverse phase, SLH: Sephadex^®^ LH-20, SPE: solid-phase extraction, THF: tetrahydrofuran, VLC: vacuum liquid chromatography, WP: whole part.

### 2.2. Structural Identification of Oxypeucedanin

Spectroscopic techniques, mainly nuclear magnetic resonance (NMR) comprising carbon-13 (13C), along with one-dimensional (1D) or 2D proton (1H) such as COSY (¹H‒¹H correlation spectroscopy), NOESY (nuclear Overhauser effect spectroscopy), HMBC (heteronuclear multiple bond correlation), and HSQC (heteronuclear single quantum coherence); mass spectrometry (MS); spectrophotometric ultra-violet (UV); and infrared (IR) have been applied in the structure elucidation of OP. Assured determination of physical properties such as melting point plays a role. Furthermore, in some studies, circular dichroism (CD) and optical rotatory power ([α]D) have been utilized to ascertain the absolute stereochemistry.

In accordance with the characteristic signals of furanocoumarins in ^1^H-NMR, which consists of a pair signals of *cis* olefinic protons linked α and β to a carbonyl (δ H 6.30 d, *J* = 10.0 Hz, H-3 and δ H 8.19 dd, *J* = 10.0, 0.5 Hz, H-4) and signals for furan olefinic protons (δ H 7.60 d, *J* = 2.0 Hz, H-2′ and δ H 6.94 dd, *J* = 2.5, 1.0 Hz, H-3′). In the ^13^C-NMR spectrum, 16 carbons can be detected, whilst regarding its being structurally prenylated, its chemical structure can be interpreted [12].

### 2.3. Analytical Investigations of Oxypeucedanin

#### 2.3.1. Identification of Oxypeucedanin in the Apiaceae Family

In general, among 24 studies identifying OP from eight species belonging to five genera of Apiaceae, 45% were carried out on *Angelica dahurica* roots. Moreover, HPLC coupled with UV and DAD detectors have been applied to analyze various soluble fractions of the plant samples specifically the methanolic extracts (12 cases). Table 2 shows the used plant species’ names, instrument, method of identification, and the OP quantities.

*Angelica* spp.

Hydro-ethanolic (96%) extracts of three Icelandic *A. archangelica* cultivars have been subjected to fingerprint HPLC-UV analysis of the major components; Accordingly, OP was qualified and quantified (0–6.45 mg/g) [71]. Moreover, the root and leaf methanolic extract was quantitatively analyzed by applying HPLC tandem MS (mass spectrometry) [72].

Considering that the root (syn. radix) part of *A. dahurica* has been described as having the richest OP content, in order to explore the optimized identification method, several analytical investigations were previously carried out to qualification and quantitation assessment of this compound. Overall, OP has mostly been identified from the methanolic extract, whereas the hydro-alcoholic fractions were also asserted. In a study reported by Fan et al. [14], reverse-phase rapid-resolution liquid chromatography (RRLC) led to OP qualification in the *A. dahurica* radix methanolic extract, possessing quantities of 0.066 to 1.45 mg/g.

In order to analyze the predominant secondary metabolites in the radix methanolic fraction of *A. dahurica*, exploiting HPLC-UV led to qualifying and quantifying OP, with quantities 0.81 µg/mL [73] and 22.30 µg/g [74]. Oxypeucedanin was quantified from the root methanolic fractions with the highest content among the studied furanocoumarins (xanthotoxin, bergapten, imperatorin, phellopterin, and isoimperatorin) at different plant growth stages, in amounts 1.5–3.0 mg/g [75]. Rising the MeOH ratio in H_2_O (60 to 100%) as the eluent system via HPLC-DAD led to measurement of the highest and lowest OP content in the root methanolic specimens dried by freezing and at 70 °C, respectively [76].

Supercritical fluid chromatography (SFC) has been utilized to analyze the methanolic partitions of the five root samples of *A. dahurica*, and OP was subsequently quantified at 1.54–2.93 g/100 g [77]. In a recent study performed by Yang et al. [78], quantitative ^1^H-NMR was exploited to examine 11 Chinese plant samples, and the OP amount ranged from 0.17 to 0.35%.

The radix hydro-ethanolic (70%) extract of *A. dahurica* has been subjected to HPLC-DAD analysis, and the OP content was recorded at 1.24 to 4.98 mg/g among 19 plant batches [79]. The same plant fraction was characterized by HPLC-UV, and OP quantities were assessed as 0.063 and 0.024% in the harvested samples from Korea and China, respectively [80]. In a rapid identification study by developing LC-NMR-MS, OP was qualified in the root ethanolic extract of *A. dahurica* [81].

Yu et al. [82] studied the bitter compounds (six coumarins) of *A. dahurica* root by using HPLC-DAD-ESI (electrospray ionization)-MS. The plant materials were prepared via boiling in H_2_O and frying in soybean oil, and OP contents were found to be 15 and 8 µg/mL, respectively.

*Ostericum koreanum*

Two samples of *O. koreanum* roots have previously been collected from different locations in South Korea, extracted with MeOH, and subjected to HPLC-UV [83]. In another experiment, a hydro-ethanolic (70%) fraction of *O. koreanum* was subjected to an HPLC-UV fingerprint measurement. The OP quantities were recorded 0.70 to 21.11% and 1.02 to 12.60% in nine and five samples harvested from Korea and China, respectively [53].

*Petroselinum crispum*

In a study carried out by Caboni et al. [84], OP was quantified at 46.04 mg/kg from the aerial part methanolic extract of parsley (*P. crispum*) via HPLC-QTOF (quadrupole and time-of-flight analyzer)-MS. HPLC-UV also has been applied in qualification and quantification assessment of the curled leaf, root, and flake parts of parsley EtOAc extracts. Consequently, OP content was recorded with a remarkable amount in the leaf of up to 102.87 µg/g [55].

*Peucedanum* spp.

In a preliminary computer-assisted qualification study accomplished by using ChromSword^®^ to construct QSRR (quantitative structure–retention relationships), the PET-soluble partition of the *P. alsaticum* fruit part has been chromatographed by means of UPLC (ultra-performance liquid chromatography), and OP was qualitatively characterized [85].

Among seven *P. ostruthium* rhizome samples collected from Austria and Germany, OP content ranged from 1.58 to 25.05 mg/g in the CH_2_Cl_2_ extracts after utilizing HPLC-DAD-MS [86]. Furthermore, OP was identified in the EtOAc root fraction by applying HPLC-DAD and HPLC-UV-ESI-MS [59].

Yrjönen et al. [87] experimented with 132 root, stem, leaf, and umbel specimens of *P. palustre* at flowering stages harvested from 43 Finland locations by means of HPLC-DAD-ESI-HR (high-resolution)-MS. Subsequently, OP was analyzed as the dominant compound of the root samples (24.3 mg/g), compared to the umbel (22.8 mg/g), stem (2.62 mg/g), and leaf (2.25 mg/g) parts. Oxypeucedanin also has been qualified in the root and umbel methanolic fractions of this species by applying HPLC-MS eluting with three solvent systems consisting of H_2_O and MeOH (gradient and isocratic) as mobile phase [72]. Nonpolar soluble fraction of the *P. palustre* root was furtherly characterized to possess OP, indicating quantities of 0.16 to 0.44 mg/100 g via HPLC-DAD on six Finnish samples [88].

*Prangos ferulacea*

The impact of three extraction methods, including Soxhlet, ultrasonic-assisted extraction (UAE), and maceration on three coumarin contents of *P. ferulacea* root was elaborated comparably. The HPLC-UV results revealed that the highest OP quantity was determined in the samples extracted via UAE, at 79.27 mg/g [89].

**Table 2:** Qualification and quantification fingerprint analysis of oxypeucedanin in different plant species.

Plant Family/Herbal Product	Plant Species	Subjected Plant Part/Soluble Extract	Analytical Instrument	Eluent System for Chromatography	Quantity	Reference
Apiaceae	*Angelica archangelica*	Fr/hydro-ethanolic (96%)	HPLC-UV	H_2_O‒MeOH [40:60 to 5:95]	0‒6.45 mg/g	[71]
		R, L/MeOH	HPLC-MS	H_2_O (1% HCO_2_H)‒MeOH [40:60]; H_2_O (0.1% HCO_2_H)‒MeOH [100:0 to 70:30, 40:60, 0:100, 100:0]	na	[72]
	*Angelica dahurica*	Radix/MeOH	RRLC	H_2_O‒MeOH [55:45, 50:50, 42:58, 36:64, 30:70, 20:80]	0.066‒1.45 mg/g	[14]
		Radix/MeOH	HPLC-UV	H_2_O (0.2% H_3_PO_4_)‒MeOH [52:48, 40:60, 55:45, 48:52, 25:75]	0.816 µg/mL (by IL-DLLME)	[73]
		Radix/MeOH	HPLC-UV	H_2_O‒MeCN [70:30, 40:60, 30:70, 40:60, 70:30]	22.30 µg/g	[74]
		R/MeOH	HPLC-DAD	H_2_O‒MeOH [40:60, 20:80, 10:90, 0:100]	1.5–3.0 mg/g	[75]
		R/MeOH	HPLC-DAD	H_2_O‒MeOH [40:60, 20:80, 10:90, 0:100]	36.95 ± 1.45 (freeze dried) 24.96 ± 0.75 (shade dried) 24.22 ± 1.75 (40 °C) 32.13 ± 1.42 (70 °C)	[76]
		R/MeOH	SFC	CO_2_‒MeOH (0.1% DEA) [100:0, 97:3, 90:10]	1.54–2.93 g/100 g (0.16‒0.77%)	[77]
		nd/MeOH	^1^H-qNMR	solvent: DMSO-d_6_	6.38–6.39 ppm (0.17–0.35%)	[78]
		Radix/hydro-ethanolic (70%)	HPLC-DAD	H_2_O (0.1% HCO_2_H)‒MeOH [95:5, 35:65, 5:95] (for qualification) H_2_O (0.1% HCO_2_H)‒MeOH [70:30, 40:60, 40, 5:95] (for quantification)	1.24–4.98 mg/g	[79]
		Radix/hydro-ethanolic (70%)	HPLC-UV	H_2_O‒MeCN [30:70]	0.063 ± 0.01 % (collected from Korea) 0.024 ± 0.02 % (collected from China)	[80]
		R/EtOH	LC-NMR-MS	H_2_O‒MeCN [40:60]	na	[81]
		R/H_2_O (for boiled sample) and soybean oil, MeOH (for fried sample)	HPLC-DAD-ESI-MS	H_2_O (0.1% HCO_2_H)‒MeOH [for boiled sample: 95:5, 75:25, 26:64, 95:5] [for fried sample: 95:5, 85:15, 77:23, 47:53, 20:80, 95:5]	15 µg/mL (for boiled sample) 8 µg/mL (for fried sample)	[82]
	*Ostericum koreanum*	R/MeOH	HPLC-UV	H_2_O‒MeCN [65:35 to 25:75]	0.57 ± 0.26 0.99 ± 0.89	[83]
		nd/hydro-ethanolic (70%)	HPLC-UV	H_2_O‒MeCN [40:60]	0.70 ± 0.02–21.11 ± 0.07% (Korean samples) 1.02 ± 0.01–12.60 ± 0.10% (Chinese samples)	[53]
	*Petroselinum crispum*	AP/MeOH	HPLC-QTOF-MS	H_2_O (0.1% HCO_2_H)‒MeCN [90:10, 60:40, 20:80, 10:90, 0:100, 90:10]	46.04 ± 5.50 mg/kg	[84]
		L, R, flake/EtOAc	HPLC-UV	cyclohexane‒isopropyl ether‒*n*-amyl alcohol [15:4:0.5]	102.87 ± 14.08 µg/g (curled L) 88.68 ± 6.04 µg/g (curled R) 88.60 ± 17.90 µg/g (flake)	[55]
	*Peucedanum alsaticum*	Fr/PET	UPLC	H_2_O‒MeCN [74:26 to 55:45]	na	[85]
	*Peucedanum ostruthium*	Rh/CH_2_Cl_2_	HPLC-DAD-MS	H_2_O (0.01% HOAc)‒MeCN (0.01% HOAc) [75:25 to 63:37 to 55:45 to 35:65 to 5:95]	1.58 ± 0.03–25.05 ± 0.11 mg/g	[86]
		R/EtOAc	HPLC-DAD (RP-C_30_) HPLC-UV-ESI-MS	H_2_O (0.1% HOAc)‒MeOH [100:0 to 0:100]	na	[59]
	*Peucedanum palustre*	R, St, L, umbel/MeOH	HPLC-DAD-ESI-HR-MS	H_2_O (0.01 M HCO_2_H)‒MeCN [100:0 to 40:60, 10:90]	24.3 ± 14.0 mg/g (in R) 2.62 ± 1.56 mg/g (in St) 2.25 ± 1.28 mg/g (in L) 22.8 ± 30.9 mg/g (in umbel)	[87]
		R, umbel/MeOH	HPLC-MS	H_2_O (1% HCO_2_H)‒MeOH [40:60] H_2_O‒MeOH (1% HCO_2_H) [71:29, 0:100], H_2_O‒MeOH (1% HCO_2_H) [100:0 to 70:30, 40:60, 0:100, 100:0]	nd	[72]
		R/NHEX	HPLC-DAD	THF‒MeCN‒MeOH‒H_2_O [3.1:35:5.4:56.5]	0.16–0.44 mg/100 g	[88]
	*Prangos ferulacea*	R/NHEX, hydro-ethanolic (95%), MeOH	HPLC-UV	H_2_O‒MeOH [30:70]	59.38 ± 0.007 mg/g (extraction with Soxhlet, NHEX) 79.27 ± 0.22 mg/g (extraction with UAE, hydro-ethanolic 95%) 55.29 ± 0.01 mg/g (extraction with maceration, MeOH)	[89]
Rutaceae	*Atalantia ceylanica*	Se/MeOH	HPLC-UV	nd	na	[90]
	*Citrus* spp.	EO	HPLC-UV	NHEX‒isopropanol [98:2]	0.7–1.65 g/L (Lemon EO from Sicily) 0.95 g/L (Lemon EO from Spain) 2.02 g/L (Lemon EO from Argentina) 0.49 g/L (Lime EO from Mexican 1) 0.96 g/L (Lime EO from Mexican 2) 0.68 g/L (Lime EO from Iran)	[91]
		EO	HPTLC	CH_2_Cl_2_‒DEE [100:3], CHCl_3_‒heptane [95:5]	na	[92]
		EO	HPLC-UV	NHEX + EtOAc (92:8)‒NHEX + EtOH (90:10)	1.55 ± 0.38 g/kg (extracted EO by Sfumatrice technology) 2.2 ± 0.41 g/kg (extracted EO by Pelatrice technology) 1.9 ± 0.45 g/kg (extracted EO by FMC technology) 0.86 ± 0.26 g/kg (extracted EO by Torchi technology)	[93]
	*Citrus aurantifolia* & *C. latifolia*	EO	HPLC-DAD	H_2_O + MeOH + THF (85:10:5)‒MeOH + THF (95:5) [100:0, 60:40, 10:90, 100:0]	4.09–10.54 g/L (*C. aurantifolia*) 0.27–10.72 g/L (*C. latifolia*)	[94]
		EO	HPLC-UV	NHEX + EtOAc (93:7)‒NHEX + EtOH (90:10) [100:0 to 5:95, 100:0]	144 mg/100 g (*C. aurantifolia*) 210–328 mg/100 g (*C. latifolia*)	[95]
	*Citrus aurantifolia* & *C. latifolia* & *C. paradisi*	EO	HPLC-DAD	H_2_O‒MeCN [70:30 to 60:40]	na	[67]
	*Citrus limon*	Wax/EO	UHPLC-DAD	H_2_O (0.1% HCO_2_H)‒MeCN [25:75, 100:0, 50:50, 30:70]	62 ± 0.8 mg/kg	[96]
		EO	HPLC-DAD	NHEX + EtOAc (92:8)‒NHEX + EtOH (90:10) [100:0 to 0:100]	89–157 mg/100 g	[66]
	*Citrus medica*	Fr/EO	HPLC-DAD	H_2_O‒MeCN [70:30, 40:60, 0:100, 70:30]	2.03–21.30 g/100 g	[97]
	*Eureka limon*	peel/EO	GC-MS	-	na	[98]
	Yuanhu zhitong (Chinese herbal drug)	Hydro-methanolic (75%)	UPLC-Q-TOF-MS	H_2_O (0.2% HCO_2_H)‒MeCN	na	[99]
		Hydro-methanolic (75%)	RRLC-QQQ	H_2_O (0.3% HCO_2_H)‒MeCN [80:20, 60:40, 20:80]	0.12–4.01 µg/g	[100]

^1^H-qNMR: quantitative ^1^H NMR, AAc: ammonium acetate, AF: ammonium format, AP: aerial part, CH_2_Cl_2_: CH2Cl2, CHCl_3_: chloroform, CO_2_: carbon dioxide, C_max_: maximum concentration, DAD: diode array detector, DEA: diethyl amine, DEE: diethyl ether, DMSO: dimethyl sulfoxide, EO: essential oil, ELSD: evaporative light-scattering detector, ESI: electrospray ionization, EtOAc: ethyl acetate, EtOH: ethanol, Fr: fruit, GC: gas chromatography, H_3_PO_4_: phosphoric acid, H_2_O: water, HCO_2_H: formic acid, HOAc: acetic acid, HR: high resolution, HPLC: high-performance liquid chromatography, IL-DLLME: ionic liquid dispersive liquid–liquid microextraction, L: leaf, LC: liquid chromatography, MeCN: acetonitrile, MeOH: methanol, MS: mass spectrometry, NHEX: *n*-hexane, na: not analyzed, NMR: nuclear magnetic resonance, PET: petroleum ether, QTOF: quadrupole and a time-of-flight analyzer, R: root, RP: reverse phase, Rh: rhizome, RRLC: rapid-resolution liquid chromatography, RRLC-QQQ: rapid-resolution liquid chromatography-triple quadrupole mass spectrometry, Se: seed, SFC: supercritical fluid chromatography, St: stem, THF: tetrahydrofuran, TNBS: trinitrobenzenesulfonic acid, UAE: ultrasound assisted extraction, UPLC: ultra-performance liquid chromatography, UV: ultraviolet-Visible.

#### 2.3.2. Characterization of Oxypeucedanin in the Rutaceae Family

As depicted in Table 2, oxypeucedanin has predominantly been qualified from the essential oils of *Citrus* spp.; however its peel, wax, and fruit parts were also rich in OP. An HPLC instrument coupled with DAD and UV as detectors was utilized as the main analytical tool to characterize its content. The OP contents were richest in the *Citrus limon* and *C. latifolia* essential oils.

*Atalantia ceylanica*

Oxypeucedanin, along with the linear furanocoumarins bergapten, xanthotoxin, heraclenin, and imperatorin, as well as two novel oximes, have previously been identified in the seed methanolic extract of *A. ceylanica* by means of HPLC-UV; however, no more information of the chromatographic conditions and OP content has been reported [90].

*Citrus* spp.

Essential oils extracted from plants of the genus *Citrus* can be considered as the richest samples among the Rutaceae family in terms of the OP occurrence. Dugo et al. [91] quantitatively and qualitatively analyzed OP content by applying HPLC-UV through an isocratic solvent mixture of NHEX–isopropanol (98:2) as the mobile phase. The highest and lowest OP concentrations were determined in lemon oil harvested from Argentina and lime oil from Mexico, at 2.02 and 0.49 g/L, respectively. HPTLC (high-performance thin-layer chromatography) also was used to analytically qualify OP in *Citrus* spp. oil, while the mobile phases were CH_2_Cl_2_–DEE (100:3) and CHCl_3_–heptane (95:5) [92].

In a similar study performed by Verzera et al. [93], four *Citrus* spp. essential oils were extracted through diverse technologies and subjected to an analytical HPLC-UV eluting with the combined solvents of NHEX–EtOAc (92:8) and NHEX–EtOH (90:10) as the mobile phases. Overall, the samples extracted with Pelatrice technology indicated the highest OP content (2.2 g/kg).

Volatile oils of *Citrus latifolia* (Persian lemon) and *C. aurantifolia* (Key lime) have further been characterized for their coumarin contents, including OP. In an analytical experiment, HPLC-DAD was employed by using a rising ratio of MeOH + THF (95:5) from 0 to 90% in H_2_O + MeOH + THF (85:10:5) to detect OP at quantities of 4.09–10.54 g/L and 0.27–10.72 g/L in the selected Key and Persian lime samples, respectively [94]. The oils of one Key and seven Persian lime samples have previously been extracted to analytically test their OP content. HPLC-UV eluting via the mixture of solvents NHEX + EtOAc (93:7) and NHEX + EtOH (90:10) from 100:0 to 5:95 led to quantifying OP contents of 144 mg/100 g and 210–328 mg/100 g in the Key and Persian lime specimens, respectively [95].

In another investigation, OP was qualified in the essential oils extracted from Key and Persian limes, and grapefruit (*C. paradisi*), and H_2_O in MeCN (70:30 to 60:40) was applied as a gradient mobile phase through an HPLC-DAD instrument [67]. Ultra-HPLC (UHPLC) coupled with DAD has previously been exploited to analyze the extracted fruit oils of *C. limon* harvested from Sicily, Italy—locally named as “Diamante citron”; whilst by increasing concentration of MeCN in H_2_O (0.1% HCO_2_H) as the mobile phase, OP was qualified and quantified (62 mg/kg) in this plant material [96]. A similar study was accomplished by hiring a gradient binary solvent system of NHEX + EtOAc (92:8) in NHEX + EtOH (90:10) via HPLC-DAD, leading to the identification of bergamottin as the major coumarin compound (160 mg/100 g) among 37 industrial cold-pressed Italian lemon oils; however, OP was also quantified with remarkable amounts of 89–157 mg/g [66].

The essential oils extracted from nine green Italian *C. medica* fruit samples also have been studied by exploiting HPLC-DAD through mobile phase H_2_O–MeCN (70:30 to 0:100) as the eluent system, and OP concentration was quantified at 2.03–21.3 g/100 g [97]. Barth et al. [98] further experimentally analyzed the *Eureka limon* peel’s essential oil in samples collected from Ivory Coast in France by application of GC-MS. Oxypeucedanin could be detected in the residue part, which was gained from the remaining materials after washing the SFC column with EtOH.

#### 2.3.3. Identification of Oxypeucedanin from Other Natural Sources

The Chinese herbal drug “Yuanhu zhitong” is combined of several medicinal herbs, mainly *Angelica dahurica* radix (baizhi) and *Corydalis* rhizome (yanhusuo). Its healing applications in the treatment of costalgia, gastralgia, dysmenorrheal, and headache have been documented in China [99].

In two experiments, its hydro-ethanolic (75%) extract was prepared and subjected to an assessment of its major components. In a study by Xu et al. [99], OP was characterized by applying UPLC-Q-TOF-MS in 15 drug samples, whilst H_2_O (0.2% HCO_2_H) and MeCN were employed in the mobile phase system. Moreover, rapid-resolution liquid chromatography-tandem triple quadrupole mass spectrometry (RRLC-QQQ) was employed to qualify and quantify the assessment of 17 compounds in 15 drug samples. Enhancing the ratio of MeCN from 20 to 80% in H_2_O (0.3% HCO_2_H) was chosen as the optimized eluent system, leading to quantify OP varying from 0.12 to 4.01 µg/g [100].

## 3. Biological Activities of Oxypeucedanin

So far, numerous preclinical biological properties of OP have been determined in vitro and in vivo. Most of these studies were focused on in vitro assessment of cytotoxic (n: 9); anti-inflammatory, antimicrobial, and antioxidant (n: 5); and antiproliferative and acetylcholinesterase inhibitory (AChE) (n: 4) activities, taking into account that due to the probable toxicity of this compound, its efficacy had not been elaborated in clinical trials yet.

In brief, OP proved good antifeedant activities against *Spodoptera littoralis* and *S. litura* larvae in the tested dosages. Moreover, it indicated potent antiallergic inflammation (histamine H1 receptor antagonist), antibiofilm activity against *Pseudomonas aeruginosa* PAO1, considerable antiproliferative effects on HTC15 (colon cancer), and UVA-irradiated B16F10 (melanoma) cell lines, in addition to high anti-influenza potencies against influenza A-H1N1 and influenza A-H9N2 (higher than ribavirin as the positive control), significant cytotoxic activity against LNCaP (androgen-sensitive human prostate adenocarcinoma) compared to finasteride, and remarkable synergistic activity with docetaxel (anticancer drug). Table S1 illustrates the comprehensive biological information of OP, including the activities, experimented media, applied assays, positive controls, and cell lines/strains.

### 3.1. Antiallergic Activity

In an in vivo study, OP showed a weak inhibitory effect on compound 48/80 (condensation products of *N*-methyl-*p*-methoxy phenylethylamine with formaldehyde)-induced histamine in mouse peritoneal, and no antiallergic activity in the analyzed dosages was observed. Nevertheless, the histamine contents at doses of 10 and 20 mg/kg were assessed 12.6 ± 1.75 and 12.2 ± 1.00 µg/100 mL of cavity fluids, respectively [26].

### 3.2. Antiarrhythmic Activity

Application of an hKv1.5 channel inhibitory assay indicated that OP prolonged the cardiac action duration of rat atrial and ventricular muscles in a dose-dependent manner. The IC_50_ determined was 76.12 ± 8.07 nM, while it had no effect on human Eag-related gene (HERG) current [16].

### 3.3. Anticonvulsant Activity

No anticonvulsant effect was recorded for OP at a concentration of 300 mg/kg, performed using maximal electroshock-induced seizure in mice model (in vivo) [101]. In another experiment on 18 coumarin derivatives, the modulation of GABA-induced chloride currents (I_GABA_) on recombinant α1β2γ2S GABAA receptors expressed in *Xenopus laevis* oocytes were analyzed. Accordingly, an EC_50_ value of 26 ± 8 μM and I_GABA_ of 550 ± 71% (at 100 μM) were measured for OP [102].

### 3.4. Antifeedant Activity

The leaf-disk bioassay has previously been utilized for evaluation antifeedant activity of OP. It revealed inhibitory effects against *Spodoptera littoralis* larvae, with ED_50_ of 41.92 ± 18.74 mg/L [103]. In a bioassay-guided isolation of antifeedant compounds from *Skimmia japonica*, the antifeedant index was calculated at 19.83 ± 6.91% at a concentration of 1 mg/mL of OP against *Spodoptera litura* larvae, in which the control group demonstrated feeding-deterrent activity, with an index of 23.62 ± 9.64% [68].

### 3.5. Antigenotoxic Activity

Marumoto et al. [104] investigated antigenotoxic impacts of several natural coumarins by exploiting Umu Chromotest™ in vitro. No genotoxic activity was detected for OP against carcinogens induced by AF-2 (furylfuramide) and MNNG (N-methyl-N’-nitro-N-nitrosoguanidine). The OP antigenotoxicity also was determined against procarcinogen of PBTA-4 (2-[2-(acetylamino)24-amino-5-methoxyphenyl]25-amino-7-bromo-4-chloro-2H-benzotriazole, at 26 µM in the presence of rat S9 mix with IC_50_ of 1.69 ± 0.28 µM; whereas on the genotoxic activation of PBTA-4 and MeIQ (2-amino-3,4-di-methylimidazo[4,5-f]quinoline) catalyzed by rat and human CYP1A1 enzymes, IC_50_ were 0.24 ± 0.08 and 30.14 ± 0.08 µM, respectively. Moreover, OP was efficient against MeIQ (0.016 µM) on the above-mentioned enzymes.

### 3.6. Anti-Inflammatory Activity

In an in vitro experiment, using a NO production assay, no anti-inflammatory activity was identified for OP in the culture medium of LPS (10 µg/mL)/IFN-*γ* (10 units/mL)-stimulated RAW 264.7 cell line (murine macrophage) [81]. In a study carried out by Li and Wu [28], OP exhibited a potent histamine H1 receptor antagonist via virtual screening by a docking program. This compound demonstrated antiallergic inflammation by reducing histamine release. Moreover, OP inhibited the secretion of tumor necrosis factor-α (TNF-α), interleukin (IL)-1β, and IL-4 in RBL-2H3 cells (rat basophilic leukemia), possessing secretion concentrations of 100, 120, and 39 pg/mL, respectively; whereas DNP-HAS (dinitrophenyl-human serum albumin) was applied as a positive control, with secretion of 160, 240, and 55 pg/mL, respectively [28].

L-*N*^G^-Nitro arginine methyl ester (L-NAME, 100 mM) has been applied as positive control in a NO production assay against lipopolysaccharide (LPS)-activated RAW 264.7 macrophage cells; OP demonstrated a moderate anti-inflammatory activity, with IC_50_ of 16.8 µg/mL [15]. Using the same method, OP showed anti-inflammatory potency (IC_50_: 57 µM) in LPS-activated mouse peritoneal macrophage cell, while the activity was lower than L-NMMA (*N*^G^-monomethyl-L-arginine) with IC_50_ 28 µM [36]. Moreover, Murakami et al. [64] illustrated a weak NO accumulation inhibitory activity of OP against RAW 264.7 macrophage cells, possessing IC_50_ of 310 µM, compared to *N*-(iminoethyl)-L-ornithine with IC_50_ value 7.9 µM, applied as the positive control.

### 3.7. Antimalarial Activity

*Plasmodium falciparum*, a unicellular protozoan parasite, is considered the deadliest species of *Plasmodium,* causing malaria in human [105]. In one study, no antimalarial effect was identified for OP against this parasite; chloroquine and artemisinin were employed as positive controls [69]. 

### 3.8. Antimicrobial Activity

Mileski et al. [38] elaborated antibacterial potency of OP against several Gram-negative and Gram-positive bacteria by using a microbroth dilution method. The highest effect was detected against *Bacillus cereus,* with MIC and MBC values of 2.00 ± 0.03 and 4.00 ± 0.06 mg/mL, respectively; whilst the antibacterial drug streptomycin exhibited 0.09 ± 0.00 and 0.37 ± 0.02 mg/mL, respectively. In the same study, antiquorum sensing and antibiofilm activities of different extracts of *Angelica pancicii* and its isolated compounds were used against *Pseudomonas aeruginosa* PAO1. The maximum diameter reduction was recorded in the presence of OP among all samples. This furanocoumarin presented a lower colony diameter of 8.66 ± 4.04 mm, compared to streptomycin and ampicillin, with 11.00 ± 1.00 and 13.33 ± 5.03 mm, respectively [38].

Oxypeucedanin revealed no antifungal activity against three plant pathogens comprising the bacteria *Xanthomonas compestris Erwinia cartovorum*, and a fungus *Sclerotinia sclerotorium* by applying a disk diffusion assay [63]. A microbroth dilution assay was used to assess antimycobacterial activity of OP isolated from dill (*Anethum graveolens*) against five microorganisms. The highest potencies were measured against *Mycobacterium smegmatis* and *M. aurum,* each with the same MIC value of 32 μg/mL, in comparison with ethambutol (positive control), possessing an MIC of 0.5 μg/mL [12]. Moreover, Tavakoli et al. [46] reported an antibacterial effect of OP (concentration: 50 μg/disk) against *Klebsiella pneumoniae* and *Shigella dysenteriae,* indicating inhibition zones of 13 mm evaluated by disk diffusion assay.

OP indicated moderate effects among the compounds isolated from *Levisticum officinale* against several bacteria pathogens. When a microbroth dilution method was applied, it revealed potencies, with MIC values of 64 and 256 against multidrug-resistant *Mycobacterium tuberculosis* and *Staphylococcus aureus*, respectively, although it exhibited weaker effects compared to the applied positive controls [70].

### 3.9. Antioxidant Activity

Oxypeucedanin isolated from five plant species has been evaluated for its ability to scavenge free radicals. A DPPH (2,2-diphenyl-1-picrylhydrazyl) assay displayed antiradical activity of OP identified in *Angelica dahurica,* with an IC_50_ value higher than 200 μg/mL [23]. This bioassay also has been utilized to evaluate the antiradical potency of OP extracted from *Ferulago subvelutina*. It exhibited a weak activity (IC_50_: 217 μg/mL) compared to BHT (butylated hydroxytoluene, IC_50_: 27 μg/mL) as the reference standard [54]. Furthermore, by applying the FRAP (ferric-reducing antioxidant power) method, OP isolated from *F. trifida* showed an effect of 9.15 ± 1.7 mm FSE (ferrous sulphate equivalents)/100 g; however, BHT indicated a greater effect of 267.2 ± 1.7 mm FSE/100 g [46].

Razavi et al. [63] reported an antiradical activity of OP with an RC_50_ value of 51.25 mg/mL, determined with DPPH assay. OP also indicated remarkable antioxidant activity among all 14 isolated compounds from *Zanthoxylum flavum*, possessing an IC_50_ of 8.3 μg/mL, compared to ascorbic acid (IC_50_: 1.4 μg/mL), when the cell-based DCFH-DA (dichloro-dihydro-fluorescein diacetate) method was employed [69].

### 3.10. Antiproliferative Activity

In a study by Kim et al. [35], the SRB (sulforhodamine B) assay was exploited to investigate in vitro cell-proliferation activity of OP against five selected cell lines. In a dose-dependent manner, the highest potency was detected on HTC15 (colon cancer cell line) with an ED_50_ of 3.4 ± 0.3 μg/mL, whilst cisplatin showed an ED_50_ of 2.2 ± 0.4 μg/mL as the positive control.

Psoralen (IC_50_ of 0.11 μM) has previously been used as positive control for assessing the in vitro antiproliferative activity against the UVA-irradiated B16F10 melanoma cell line, and OP demonstrated an effect, with an IC_50_ 0.22 μM. These promising findings can be considered for further OP application in the treatment of photo-dermatosis disorders. Moreover, in this study, OP’s impact on the aforementioned cell line was evaluated in vivo in a mouse model as well. Interestingly, OP reduced tumor volume from 2200 (in control group) to 500 mm^3^ after 20 days of treatment (0.5 mg/kg), along with decreasing the tumor weight from 2000 mg to 250 mg (at 0.5 mg/kg of administration) [27].

Five cancer cell lines comprising MDA-MB-231 (breast cancer cell), T47D (breast cancer cell), SNU638 (stomach cancer cell), SK-Hep-1 (human hepatoma cell), and A549 (lung cancer cell) were subjected to assess proliferation activity of OP by utilization of an SRB assay. Among them, OP showed the highest potency against SK-Hep-1 cells, with an IC_50_ of 32.4 μM; however, it was lower than etoposide as the control (IC_50_: 0.3 μM). The authors asserted that this activity may be correlated with the induction of G2/M phase cell-cycle arrest and upregulation of the p53/MDM2/p21 axis in SK-HEP-1 hepatoma cells by OP after 72 h of treatment [19].

In our previous study, doxorubicin, a chemotherapy medication, has been applied as the positive control to study the antiproliferative effect of the isolated furanocoumarins from *Ducrosia anethifolia* via an MTT assay. Moderate effects of OP were observed against PAR (L5178Y mouse T-cell lymphoma) and MDR (ABCB1-expressing L5178Y) cell lines, possessing IC_50_s of 25.98 and 28.89 µM, respectively; while doxorubicin showed stronger potencies, with IC_50_s of 0.05 and 0.46 µM, respectively. Oxypeucedanin also exhibited a slight synergistic effect with doxorubicin when using a checkerboard combination assay against the MDR cell line with CI (combination index), with an ED_50_ of 0.85 ± 0.07 at 1:50 ratio [41].

### 3.11. Antiviral Activity

Oxypeucedanin isolated from *Angelica dahurica* notably exhibited higher anti-influenza activity compared to the control drug. In this study, administration of OP (20 μM, after 2 h) considerably decreased the levels of viral proteins NP (nucleoprotein) and NA (neuraminidase) in the treated cells to less than 20% via inhibition of their synthesis. The EC_50_ values were measured at 5.98 ± 0.71 and 4.52 ± 0.39 μM against influenza A-H1N1 and influenza A-H9N2, respectively; while ribavirin possessed 6.29 ± 0.89 and 6.13 ± 0.19 μM, respectively [20]. Nonetheless, in accordance with Shokoohinia et al. [61], no anti-HSV (antiherpes) activity was detected for OP against the Vero cell line (African monkey kidney).

### 3.12. Calcium Antagonistic Activity

Calcium antagonistic activity of 15 compounds isolated from *A. dahurica* has previously been evaluated by measuring the depolarization-induced ^45^Ca^2+^ uptake in clonal GH_4_C_1_ rat pituitary cells, and OP showed an inhibitory effect of 43 ± 4.3% at a concentration of 20 μg/mL [13].

### 3.13. Cytotoxic Activity

An MTT assay has been used to evaluate cytotoxic activities of certain coumarins on tacrine-induced cytotoxicity in Hep-G2 cells. OP indicated no cytotoxicity, while the other studied coumarins (imperatorin, byakangelicin, and byakangelicol) were active. The EC_50_ of OP was found to be 286.7 ± 6.36 µM, a lower effect than silybin (EC_50_: 69.0 ± 3.4 µM) as the control [18]. Moderate cytotoxic activities of OP were determined by means of an MTT assay (in vitro) against four tested cancer cell lines: L1210 (murine leukemia), HL-60 (human leukemia), K562 (human leukemia), and B16F10 (murine melanoma). Although two of OP’s hydrated derivatives, namely pangelin and oxypeucedanin hydrate acetonide, indicated the highest effects, OP with an IC_50_ of 27.5 µg/mL showed weaker cytotoxicity than Adriamycin (syn. doxorubicin, IC_50_: 2.8 µg/mL) against the HL-60 cell line [24].

In a further study, OP isolated from *A. koreanum* displayed an equal cytotoxic activity with finasteride (control drug), both possessing IC_50_ values >20 µg/mL against the LNCaP cell line (androgen-sensitive human prostate adenocarcinoma) [37]. Nevertheless, doxorubicin as the chemotherapy drug showed higher in vitro cytotoxic effects on three experimented cancer cells, and OP was the most potent furanocoumarin derivative against parent (PAR, IC_50_: 40.33 ± 0.63 µM)and multidrug-resistant (MDR, IC_50_: 66.68 ± 0.00 µM) cells, and had slight toxicity on normal murine fibroblasts (NIH/3T3, IC_50_: 57.18 ± 3.91 µM) [41]. The highest OP cytotoxicity was recorded against the A-549 cell line (lung carcinoma), with an IC_50_ of 0.80 mM among four tested cells, compared to the positive control (tamoxifen, IC_50_: 0.017 mM) [46].

The human prostate carcinoma cell (DU145) has been used to assess OP’s cytotoxicity. Results demonstrated that this compound significantly inhibited cell growth, with a maximum of 93.3% (at 100 µM after 72 h treatment) by G2-M cell cycle arrest, and induced apoptotic cell death up to 50% (at 100 µM after 72 h treatment) [52]. By applying an MTT assay, Choi et al. [51] exhibited that OP was not able to reduce cell viability up to 100 μM for 24 h against mice neuroblastoma neuro-2A cells. A higher CC_50_ (50% cytotoxic concentration) for OP (272.6 ± 6.9 μM) asserted lower effectiveness of this naturally occurring compound in comparison with the anticancer drug etoposide (CC_50_: 46.9 ± 10.5 μM).

Using the same assay, cytotoxicity of OP was determined on the HeLa cell line, possessing an IC_50_ value of 314 μg/mL [63]. Cytotoxicity of OP isolated from *Zanthoxylum flavum* also has been investigated against the HL-60 (IC_50_: 8.9 µg/mL) and Vero cell lines (no activity) [69]. According to Dong et al. [106], multidrug resistance of the chemotherapeutic drugs comprising vincristine sulphate (VCR) and docetaxel (DTX) were mainly caused by P-glycoprotein. An MTT assay was applied to evaluate permeability (apical to basolateral side) of OP to Mardin Darby canine kidney (MDCK) cells. As a result, OP drastically increased the apparent permeability (P_app_) of VCR (264.46 µmol/L) from 0.37 ± 0.04 × 10^-6^ to a maximum of 0.52 ± 0.04 × 10^-6^ cm/s at an OP concentration of 76.30 µmol/L.

### 3.14. Enzyme Inhibitory Activity

The reversible inhibitors of AChE (acetylcholinesterase enzyme) are being utilized generally in the treatment of neurodegenerative disorders, including Alzheimer’s disease (AD). Inhibition of brain AChE is considered as the major therapeutic strategy for treating this ailment [107]. So far, AChE inhibitory activity of many natural products has been investigated to develop novel drug candidates [108,109,110,111]. Oxypeucedanin demonstrated a dose-dependent AChE inhibitory activity in the study of Kim et al. [17]. In this study, an Ellman assay was applied to analyze its slight effect (IC_50_: 89.1 μM) in comparison with berberine (IC_50_: 2.9 μM) as the control drug. In another study, OP indicated a weak anti-AChE activity, with an IC_50_ of 69.3 ± 1.1 µg/mL, while eserine had an IC_50_ of 0.51 ± 0.03 µg/mL as the positive control [19]. Moreover, by utilizing the Ellman method, OP revealed the strongest AChE (19.36 ± 1.87%) and BChE (butyrylcholinesterase enzyme, 36.89 ± 1.23%) inhibitory effects among the compounds isolated from *Angelica purpurascens,* including stigmasterol, β-sitosterol, and bergapten [39].

Furthermore, inhibitory activity of OP against BACE1 (β-secretase) as an effective target in AD treatment has been carried out in vitro. OP was determined as the weakest compound among the other four isolated coumarins (isoimperatorin, imperatorin, (+)-byakangelicol, and (+)-byakangelicin), with an IC_50_ value of 359.2 ± 1.23 µM, whereas the control (Lys-Thr-Glu-Glu-Ile-Ser-Glu-Val-Asn-(statine)-Val-Ala-Glu-Phe-OH) possessed an IC_50_ of 0.2 ± 0.01 µM [22].

Oxypeucedanin isolated from *A. koreana* exhibited a weak inhibitory activity against 5α-reductase type I, an enzyme responsible for changing androgen testosterone into its activated form, dihydrotestosterone (DHT) [112]. This compound, with an IC_50_ higher than 20 µg/mL, demonstrated inactivity against LNCaP (androgen-sensitive human prostate adenocarcinoma) cells, although an IC_50_ of 19.8 µg/mL was recorded for finasteride as the control drug [37].

Recently, Karakaya et al. [39] studied the antilipid peroxidation activity of OP in vitro, and a moderate effect was identified, with an IC_50_ of 91.27 µg/mL. Oxypeucedanin also was evaluated for its abilities in molecular docking. The results exhibited dock scores of −7.52 and −4.23 kcal/mol against AChE (1EVE) and BChE (1P01), respectively. Moreover, OP suppressed the cytosolic isoenzyme of human carbonic anhydrase (hCA) I and II, with IC_50_s of 6.72 ± 0.98 and 5.29 ± 0.98 µM, respectively. In another investigation, OP demonstrated a good affinity to InhA (2-trans-enoyl-ACP reductase) enzyme, possessing a docking score of −7.764 kcal/mol, whilst the utilized control isoniazid indicated a docking score of −6.013 kcal/mol [70].

### 3.15. Insecticidal Activity

Oxypeucedanin possessed a moderate larvicidal effect against *Anopheles stephensi*, one of the main malaria vectors, with lethal concentrations causing 50% (LC_50_) and 90% (LC_90_) of mortality by 116.54 and 346.41 ppm, respectively [45].

### 3.16. Phytotoxic Activity

A lettuce assay has been applied to assess OP phytotoxicity in two studies. Sbai et al. [56] reported that OP isolated from *Petroselinum crispum* indicated a good effect, in particular on the inhibition of the *Lactuca sativa* (lettuce, 0.06 g/L) shoot length, at −24.37 ± 7.63% of control. However, this secondary metabolite showed effective impacts on the total seed germination (93.33 ± 7.63%) and root length (26.41 ± 15.88%). In a similar study, using the abovementioned method, OP showed remarkable phytotoxic activity in inhibition of seed germination (IC_50_: 0.21 mg/mL), and growth inhibitions of the shoot (IC_50_: 0.59 mg/mL) and root (IC_50_: 0.62 mg/mL) of lettuce at concentrations over 0.1 mg/mL [63].

## 4. Pharmacokinetic Analysis of Oxypeucedanin

As shown in Table 3, so far, seven studies have reported pharmacokinetic aspects of OP in living organisms. In an analysis performed by Chen et al. [113], the tissue distribution of OP was assessed in rat’s lung and liver after oral administration (0.46 g/kg). UHPLC coupled with an evaporative light-scattering detector (UHPLC-ELSD) was analytically applied via an enhancing ratio of MeCN (0.1% formic acid) from 5 to 95% in ammonium format. The OP concentrations were subsequently recorded at 0.05 and 0.013 µg/g in the lung and liver tissues, respectively.

An isocratic eluting solvent system consisting of H_2_O (0.1% HCO_2_H) in MeOH (28:72) was developed in HPLC-ESI-MS to determine the OP content in rat’s bile and urine by oral administration of the *Angelica dahurica* radix extract. OP was excreted in the rat’s bile and urine at 0.17 and 0.082%, respectively [114].

Hwang et al. [115] analytically quantified the OP content in *A. dahurica* (6.67%) by exploiting UHPLC-MS/MS eluting with MeCN (35 to 60%) in H_2_O. Moreover, maximum concentrations of OP were elaborated after administration of the plant radix to normal and colitis-induced rats. In the case of normal rats, the OP content was increased from 38.5 to 101.2 ng/mL by raising of the plant extract doses from 0.5 to 1.0 g/kg, respectively. Notably, OP concentration also was increased in TNBS (2,4,6-trinitrobenzene sulfonic acid)-treated rats from 29.0 to 61.2 ng/mL by administration of the plant extract at 0.5 and 1.0 g/kg, respectively.

A sensitive gas chromatography-mass spectrometry (GC-MS) method was previously developed for pharmacokinetic assessment of eight coumarins of *A. dahurica*. After OP oral administration (10 mg/kg) to rats, its maximum concentration reached 0.46 ± 0.01 µg/mL within 0.51 h [116]. In another experiment, an eluting gradient solvent system comprising H_2_O−MeOH (60:40 to 10:90) was established using an HPLC-ESI-MS/MS instrument for the pharmacokinetic study of OP. The findings revealed that *Glehnia littoralis* contained 19.0 µg/mL of OP, whereas after 72 h of oral administration, 13 and 18 ng were recorded in the rat’s urine and bile, respectively [117].

After single oral administration of 4.5 g/kg of *A. dahurica* extract containing 5.2 mg/kg OP, its maximum concentration was determined at 111 ± 25 ng/mL in the rat’s plasma after 12 h. In this study, LC-MS/MS eluting with MeCN (30 to 70%) in H_2_O (0.1% HCO_2_H) was employed [118]. In another pharmacokinetic study, an LC-MS/MS method (mobile phase: H_2_O−MeCN 65:35 to 15:85) was established to evaluate OP’s impact on docetaxel (a cancer medication) absorption in the rat plasma model. The results demonstrated that OP remarkably increased its absorption; the maximum concentrations of docetaxel in the presence and absence of OP were determined to be 118.40 ± 10.93 and 178.80 ± 8.81 µg/L after 163.80 and 104.60 min of administration, respectively [106].

## 5. Conclusions and Perspectives

Oxypeucedanin has been abundantly identified in several species belonging to the Apiaceae and Rutaceae families. Notably, the root part of *Angelica dahurica* has been characterized as the richest natural source of this compound. Along with various reported preclinical bioactivity assessments, OP represented significant antifeedant, antiallergic, antibiofilm, antiproliferative, anti-influenza, and cytotoxic characteristics. However, no clinical study on OP has been carried out; toxicological studies could support its safety evaluation.

Regarding the phototoxic activities of some linear furanocoumarins, majorly psoralen, bergapten, and xanthotoxin [119], more investigations focusing on the effect and toxicity of OP could be of interest. Until now, several studies have reported the adverse effects of some furanocoumarins that are caused by undesirable interactions with certain drugs, particularly furanocoumarins rich in grapefruit [9], but no similar study has been performed to analyze OP, and indeed this can also be considered a futuristic research aim.

In accordance with the literature, further bioassay experiments on OP and its optimized semisynthesized derivatives may lead to developing promising prospective drug candidates.

## Figures and Tables

**Table 3:** Pharmacokinetic studies of oxypeucedanin.

Plant Species/Purchased	Analytical Instrument	Method of Assessment	Concentration in Living Organisms	Reference
*Angelica dahurica*	UHPLC‒ELSD	AF (0.1% HCO_2_H)‒MeCN (0.1% HCO_2_H) [95:5, 86:14, 78:22, 70:30, 66:34, 62:38, 56:44, 16:84, 5:95, 95:5]	C: 0.05 µg/g in lung C: 0.013 µg/g in liver	[113]
	HPLC–ESI–MS	H_2_O (0.1% HCO_2_H)‒MeOH [28:72]	0.177% (in bile) ‒ 0.082% (in rat urine)	[114]
	UHPLC-MS/MS	H_2_O (0.1% HCO_2_H)‒MeCN [65:35, 40:60]	6.67% C_max_: 38.5 ± 1.6 ng/mL at 0.5 g/kg (in normal rats) C_max_: 101.2 ± 21.2 ng/mL at 1.0 g/kg (in normal rats) C_max_: 29.0 ± 4.0 ng/mL at 0.5 g/kg (in TNBS-treated rats) C_max_: 61.2 ± 11.9 ng/mL at 1.0 g/kg (in TNBS-treated rats)	[115]
	GC-MS	-	C_max_: 0.46 ± 0.01 µg/mL T_max_: 0.51 h	[116]
	LC-MS/MS	H_2_O (0.1% HCO_2_H)‒MeCN [65:35, 53:47, 15:85, 65:35]	C_max_: 111 ± 25 ng/mL at 4.5 g/kg extract/5.2 mg/kg OP (in rat plasma) T_max_: 12 ± 0 h	[118]
*Glehnia littoralis*	HPLC-ESI-MS/MS	H_2_O (1 mmol AAc)‒MeOH [60:40, 10:90, 60:40]	19.0 µg/mL (in plant sample) 13 ng (in rat urine after 72 h oral administration) 18 ng (in rat bile after 72 h oral administration)	[117]
Purchased	LC-MS/MS	H_2_O (0.1% HCO_2_H)‒MeCN [70:30, 52:48, 30:70, 70:30]	C_max_: 118.40 ± 10.93 µg/L (docetaxel in rat plasma) C_max_: 178.80 ± 8.81 µg/L (OP + docetaxel in rat plasma) T_max_: 163.80 ± 11.82 min (docetaxel) T_max_: 104.60 ± 11.68 min (OP + docetaxel)	[106]

AAc: Ammonium acetate, AF: ammonium format, C: concentration, C_max_: maximum concentration, ELSD: evaporative light-scattering detector, ESI: electrospray ionization, GC-MS: gas chromatography-mass spectrometry, H_2_O: water, HCO_2_H: formic acid, HPLC: high-performance liquid chromatography, LC-MS: liquid chromatography-mass spectrometry, MeCN: acetonitrile, MeOH: methanol, MS: mass spectrometry, OP: oxypeucedanin, T_max_: time to reach the maximum concentration, TNBS: 2,4,6-trinitrobenzene sulfonic acid, UHPLC: ultra-high-performance liquid chromatography.

## Supplementary Materials

The following are available online at https://www.mdpi.com/article/10.3390/plants10081577/s1, Table S1: Biological activities of oxypeucedanin.
