# Supplementary material for: Oxypeucedanin: Chemotaxonomy, Isolation, and Bioactivities
Source: Plants (Basel). 2021 Jul 30;10(8):1577. doi: 10.3390/plants10081577 (PMC8401860; doi:10.3390/plants10081577)
Supplement: Supplementary file 1 [file plants-10-01577-s001.zip › plants-1286256-supplementary.pdf]

Table S1. Biological activities of oxypeucedanin.

Activity	Isolated from plant species/purchased	Measured activity	Assay	Experimented media	Positive control/activity	Cell line/strain	Reference
Anti-allergic	*Angelica dahurica*	HC: 12.6 ± 1.75 µg/100 mL cavity fluids (at 10 mg/kg)<br>HC: 12.2 ± 1.00 µg/100 mL cavity fluids (at 25 mg/kg)	Measuring of histamine content	in vivo/mice	nd/142.6%	-	[26]
Antiarrhythmic	*Angelica dahurica*	IC_{50}: 76.12 ± 8.07 nM	hKv1.5 channel inhibitory	ex vivo/mice	-	clonal mouse *Ltk-*	[16]
Anticonvulsant	*Peucedanum ostruthium*	no activity (at 300 mg/kg)	maximal electroshock-induced seizure	in vivo/mice	-	-	[101]
	purchased	EC_{50}: 26 ± 8 µM<br>I_{GABA}: 550 ± 71% (at 100 µM)	I_{GABA}: GABA-induced chloride current modulation	ex vivo/frog	-	ovary	[102]
Antifeedant	purchased	ED_{50}: 41.92 ± 18.74 mg/L	leaf-disk bioassay	in vitro	-	*Spodoptera littoralis* larvae	[103]
	Skimmia japonica	AI: 19.83 ± 6.91% (at 1 mg/mL)	leaf-disk bioassay	-	AI: 23.62 ± 9.64%	*Spodoptera litura* larvae	[68]
Antigenotoxic	*Angelica dahurica*	No activity (on AF-2- and MNNG-induced)<br><br>IC_{50}: 1.69 ± 0.28 µM (on 26 µM PBTA-4, rat S9 mix)<br>IC_{50}: 0.24 ± 0.08 µM (on 26 µM PBTA-4, rat CYP1A1)<br>IC_{50}: 30.14 ± 0.08 µM (on 26 µM PBTA-4, Human CYP1A1)<br><br>IC_{50}: 2.49 ± 0.13 µM (on 0.016 µM MeIQ, rat S9 mix)	Umu Chromotest	in vitro	-	-	[104]

		IC_{50}: 0.96 ± 0.09 μM (on 0.016 μM MelQ, Rat CYP1A2)<br>IC_{50}: 4.39 ± 0.32 μM (on 0.016 μM MelQ, Human CYP1A1)					
Anti-inflammatory	*Angelica dahurica*	No activity	NO production assay	in vitro	Dexamethasone (0.1 mM)	RAW 264.7 cell	[81]
	Angelica dahurica	C: 100 pg/mL (secretion of TNF-α)<br>C: 120 pg/mL (secretion of IL-1β)<br>C: 39 pg/mL (secretion of IL-4)<br>C: 4.5 (on activation of nuclear factor-κB)	Luciferase assay		DNP-HSA<br>C: 160 pg/mL (secretion of TNF-α)<br>C: 240 pg/mL (secretion of IL-1β)<br>C: 55 pg/mL (secretion of IL-4)<br>C: 7 (on activation of nuclear factor-κB)	RBL-2H3	[28]
	Angelica dahurica	IC_{50}: 16.8 μg/mL	NO production assay/MTT	In vitro	L-NAME (100 mM)	Raw 264.7 macrophage cell	[15]
	Angelica furcijuga	IC_{50}: 57 μM	NO production assay/MTT assay	In vitro	L-NMMA<br>IC_{50}: 28 μM	lipopolysaccharide-activated mouse peritoneal macrophages	[36]
	Citrus hystrix	IC_{50}: 310 μM	NO production assay/MTT	In vitro	*N*-(iminoethyl)-L-ornithine (L-NIO)<br>IC_{50}: 7.9 μM	RAW 264.7 macrophage cell	[64]
Antimalarial	*Zanthoxylum flavum*	No activity	96-well microplates assay	In vitro	Chloroquine<br>IC_{50}: 15.5 ng/mL on D6 clone<br>IC_{50}: 170 ng/mL on W2 clone<br><br>Artemisinin<br>IC_{50}: 10.3 ng/mL on D6 clone<br>IC_{50}: 6.3 ng/mL on W2 clone	*Plasmodium falciparum*	[69]
Antimicrobial	*Angelica pancicii*	MIC: 2.00 ± 0.03 mg/mL<br>MIC: 16.00 ± 0.05 mg/mL<br>MIC: 4.00 ± 0.04 mg/mL<br>MIC: 4.00 ± 0.01 mg/mL	Microbroth dilution	In vitro	Streptomycin<br>MIC: 0.09 ± 0.00 mg/mL<br>MIC: 0.17 ± 0.02 mg/mL<br>MIC: 0.17 ± 0.01 mg/mL<br>MIC: 0.04 ± 0.00 mg/mL	*Bacillus cereus*<br>*Mucilaginibacter flavus*<br>*Listeria monocytogenes*<br>*Staphylococcus aureus*	[38]

	MIC: 4.00 ± 0.08 mg/mL<br>MIC: 16.00 ± 0.02 mg/mL<br>MIC: 16.00 ± 0.07 mg/mL<br>MIC: 4.00 ± 0.05 mg/mL<br><br>MBC: 4.00 ± 0.06 mg/mL<br>na<br>MBC: 16.00 ± 0.09 mg/mL<br>MBC 16.00 ± 0.05 mg/mL<br>MBC: 8.00 ± 0.02 mg/mL<br>na<br>na<br>MBC: 16.00 ± 0.07 mg/mL			MIC: 0.17 ± 0.04 mg/mL<br>MIC: 0.17 ± 0.00 mg/mL<br>MIC: 0.26 ± 0.01 mg/mL<br>MIC: 0.17 ± 0.00 mg/mL<br><br>MBC: 0.37 ± 0.02 mg/mL<br>MBC: 0.37 ± 0.00 mg/mL<br>MBC: 0.49 ± 0.03 mg/mL<br>MBC: 0.37 ± 0.02 mg/mL<br>MBC: 1.24 ± 0.00 mg/mL<br>MBC: 0.49 ± 0.03 mg/mL<br>MBC: 0.74 ± 0.07 mg/mL<br>MBC: 0.49 ± 0.03 mg/mL	*Pseudomonas aeruginosa*<br>*Escherichia coli*<br>*Enterobacter cloacae*<br>*Salmonella typhimurium*<br><br>*Bacillus cereus*<br>*Mucilaginibacter flavus*<br>*Listeria monocytogenes*<br>*Staphylococcus aureus*<br>*Pseudomonas aeruginosa*<br>*Escherichia coli*<br>*Enterobacter cloacae*<br>*Salmonella typhimurium*	
Angelica pancicii	COD: 8.66 ± 4.04 mm<br>BF: 10.60 ± 0.53% (0.5 MIC)<br>BF: 49.46 ± 0.93% (0.25 MIC)	nd	nd	Streptomycin<br>COD: 11.00 ± 1.00 mm<br>BF: 69.16 ± 0.65% (0.5 MIC)<br>BF: 56.46 ± 0.46% (0.25 MIC)<br>BF: 92.16 ± 0.37% (0.125 MIC)<br><br>Ampicillin<br>COD: 13.33 ± 5.03 mm<br>BF: 49.40 ± 0.46% (0.5 MIC)<br>BF: 70.97 ± 0.36% (0.25 MIC)<br>BF: 88.36 ± 0.42% (0.125 MIC)	*Pseudomonas aeruginosa* PAO1	[38]
Prangos uloptera	No activity	Disc diffusion	In vitro	Gentamicin	*Xanthomonas campestris*<br>*Erwinia cartovorum*<br>*Sclerotinia sclerotorium*	[63]
Anethum graveolens	MIC: 128 µg/mL<br>MIC: 64 µg/mL<br>MIC: 32 µg/mL<br>MIC: 32 µg/mL<br>na	Microbroth dilution	In vitro	Ethambutol<br>MIC: 4 µg/mL<br>MIC: 4 µg/mL<br>MIC: 0.5 µg/mL<br>MIC: 0.5 µg/mL<br>MIC: 128 µg/mL	*Mycobacterium fortuitum* (ATCC-6841)<br>*Mycobacterium phlei* (ATCC-11758)<br>*Mycobacterium aurum* (PI-104482)<br>*Mycobacterium smegmatis* (ATCC-14468)<br>*Mycobacterium abscessus* (ATCC-9977)	[12]
Ferulago trifida		Disc diffusion	In vitro	Gentamicin (at 10 µg/disc)		[46]

		IZ: 13 mm			IZ: 22 mm	*Klebsiella pneumoniae*	
		No activity			IZ: 21 mm	*Pseudomonas aeruginosa*	
		No activity			IZ: 18 mm	*Staphylococcus epidermidis*	
		No activity			IZ: 21 mm	*Bacillus subtilis*	
		No activity			IZ: 20 mm	*Escherichia coli*	
		No activity			IZ: 21 mm	*Salmonella paratyphi A*	
		IZ: 13 mm			IZ: 35 mm	*Shigella dysenteriae*	
		MIC: 500 µg/mL	Microbroth dilution		MIC: 250 µg/mL	*Klebsiella pneumoniae*	
		No activity			MIC: 500 µg/mL	*Pseudomonas aeruginosa*	
		No activity			MIC: 500 µg/mL	*Staphylococcus epidermidis*	
		No activity			MIC: 500 µg/mL	*Bacillus subtilis*	
		No activity			MIC: 500 µg/mL	*Escherichia coli*	
		No activity			MIC: 500 µg/mL	*Salmonella paratyphi A*	
		MIC: >500 µg/mL			MIC: 500 µg/mL	*Shigella dysenteriae*	
			Disc diffusion		Rifampin (5 µg/disc)		
					IZ: 7 mm	*Klebsiella pneumoniae*	
					IZ: 10 mm	*Pseudomonas aeruginosa*	
					IZ: 8 mm	*Staphylococcus epidermidis*	
					IZ: 13 mm	*Bacillus subtilis*	
					IZ: 11 mm	*Escherichia coli*	
					No activity	*Salmonella paratyphi A*	
					IZ: 40 mm	*Shigella dysenteriae*	
			Microbroth dilution		MIC: 250 µg/mL	*Klebsiella pneumoniae*	
					MIC: 250 µg/mL	*Pseudomonas aeruginosa*	
					MIC: 250 µg/mL	*Staphylococcus epidermidis*	
					MIC: 15 µg/mL	*Bacillus subtilis*	
					MIC: 500 µg/mL	*Escherichia coli*	
					No activity	*Salmonella paratyphi A*	
					MIC: 250 µg/mL	*Shigella dysenteriae*	
	Levisticum officinale	MIC: 64 µg/mL	Microbroth dilution	In vitro	Isoniazid MIC: 4 µg/mL	*Mycobacterium tuberculosis*	[60]
					Cefixime		
		MIC: 256 µg/mL	Microbroth dilution	In vitro	MIC: 2 µg/mL	*Staphylococcus aureus*	[60]
		MIC: 256 µg/mL			MIC: 4 µg/mL	*Escherichia coli*	

		MIC: 256 μg/mL<br>MIC: >256 μg/mL<br>MIC: >256 μg/mL			MIC: 16 μg/mL<br>MIC: 64 μg/mL<br>-<br><br>Chloramphenicol<br>MIC: 8 μg/mL<br>MIC: 32 μg/mL<br>MIC: 64 μg/mL<br>MIC: >256 μg/mL<br>MIC: >256 μg/mL	*Enterococcus faecium*<br>*Pseudomonas aeruginosa*<br>methicillin-resistant *S. aureus* (MRSA)<br><br><br>*Staphylococcus aureus*<br>*Escherichia coli*<br>*Enterococcus faecium*<br>*Pseudomonas aeruginosa*<br>*methicillin-resistant S. aureus* (MRSA)	
Antioxidant	*Angelica dahurica*	IC_{50}: >200 μg/mL	DPPH	In vitro	nd	nd	[23]
	Ferulago subvelutina	IC_{50}: 217 μg/mL	DPPH	In vitro	BHT<br>IC_{50}: 27 μg/mL	-	[49]
	Ferulago trifida	9.15 ± 1.7 mm FSE/100 g	FRAP	In vitro	BHT<br>267.2 ± 4.2 mmol FSE/100 g	-	[46]
	Prangos uloptera	RC_{50}: 51.25 mg/mL	DPPH	In vitro	nd	nd	[63]
	Zanthoxylum flavum	IC_{50}: 8.3 μg/mL	cell-based DCFH-DA	In vitro	Ascorbic acid<br>IC_{50}: 1.4 μg/mL	-	[69]
Antiproliferative	*Angelica dahurica*	ED_{50}: 9.5 ± 0.3 μg/mL<br>ED_{50}: 19.3 ± 0.3 μg/mL<br>ED_{50}: 16.5 ± 0.2 μg/mL<br>ED_{50}: 16.1 ± 0.5 μg/mL<br>ED_{50}: 3.4 ± 0.3 μg/mL	SRB	In vitro	Cisplatin<br>ED_{50}: 1.4 ± 0.1 μg/mL<br>ED_{50}: 0.9 ± 0.3 μg/mL<br>ED_{50}: 0.8 ± 0.2 μg/mL<br>ED_{50}: 0.9 ± 0.3 μg/mL<br>ED_{50}: 2.2 ± 0.4 μg/mL	A549 (non-small cell lung)<br>SK-OV-3 (ovary cancer cell line)<br>SK-MEL-2 (melanoma cancer cell line)<br>XF498 (central nervous system cell)<br>HCT15 (colon cancer cell line)	[35]
	Angelica dahurica	IC_{50}: 0.22 μM<br><br>TV: 500 mm^3 (at 0.5 mg/kg after 20 days)<br>TV: 900 mm^3 (at 1.0 mg/kg after 20 days)	-	In vitro<br><br>In vivo/mice	Psoralen<br>IC_{50}: 0.11 μM<br><br>TV: 2200 mm^3	UVA-irradiated B16F10 melanoma	[27]

		TW: 250 mg (at 0.5 mg/kg)<br>TW: 600 mg (at 1.0 mg/kg)			TW: 2000 mg		
	Angelica dahurica	IC_{50}: 50.8 μM<br>IC_{50}: 95.5 μM<br>IC_{50}: 50.4 μM<br>IC_{50}: 32.4 μM<br>IC_{50}: 46.3 μM	SRB	In vitro	Etoposide<br>IC_{50}: 0.3 μM	MDA-MB-231 (breast cancer cell)<br>T47D (breast cancer cell)<br>SNU638 (stomach cancer cell)<br>SK-Hep-1 (human hepatoma cell)<br>A549 (lung cancer cell)	[21]
	Ducrosia anethifolia	IC_{50}: 25.98 ± 1.27 μM<br>IC_{50}: 28.89 ± 0.73 μM	MTT	In vitro	Doxorubicin<br>IC_{50}: 0.054 ± 0.005 μM<br>IC_{50}: 0.468 ± 0.065 μM	PAR cell line<br>MDR cell line	[41]
Antiviral	*Angelica dahurica*	EC_{50}: 5.98 ± 0.71 μM<br>EC_{50}: 4.52 ± 0.39 μM	nd	In vitro	Ribavirin<br>EC_{50}: 6.29 ± 0.89 μM<br>EC_{50}: 6.13 ± 0.19 μM	influenza A - H1N1<br>influenza A - H9N2	[20]
	Prangos ferulacea	No activity	nd	In vitro	nd	Vero (African monkey kidney cell)	[61]
Calcium antagonistic	*Angelica archangelica*	I: 43 ± 4.3% (at 20 μg/mL)	$^{45}Ca^{2+}$ - uptake	rat	Verapamil<br>IC_{50}: 2.0 ± 0.2 μg/mL	Clonal pituitary GH_4C_1 cells	[13]
Cytotoxic	*Angelica dahurica*	EC_{50}: 286.7 ± 6.36 μM	MTT	In vitro	Silybin<br>EC_{50}: 69.0 ± 3.4 μM	Hep G2 cell line	[18]
	Angelica dahurica	IC_{50}: >30 μg/mL<br>IC_{50}: 27.5 μg/mL<br>IC_{50}: >30 μg/mL<br>IC_{50}: >30 μg/mL	MTT	In vitro	Doxorubicin (syn. Adriamycin)<br>IC_{50}: 0.8 μg/mL<br>IC_{50}: 2.8 μg/mL<br>IC_{50}: 1.4 μg/mL<br>IC_{50}: 0.9 μg/mL	L1210 (murine leukemia cancer cell)<br>HL-60 (human leukemia cancer cell)<br>K562 (human leukemia cancer cell)<br>B16F10 (murine melanoma cancer cell)	[24]
	Angelica koreanum	IC_{50}: >20 μg/mL	MTT	In vitro	Finasteride<br>IC_{50}: >20 μg/mL	LNCaP cell	[37]
	Ducrosia anethifolia	IC_{50}: 40.33 ± 0.63 μM<br>IC_{50}: 66.68 ± 0.0 μM<br>IC_{50}: 57.18 ± 3.91 μM	MTT	In vitro	Doxorubicin<br>IC_{50}: 0.377 ± 0.02 μM<br>IC_{50}: 7.152 ± 0.35 μM<br>IC_{50}: 5.71 ± 0.50 μM	PAR cell line<br>MDR cell line<br>NIH/3T3 cell line	[41]
	Ferulago trifida	IC_{50}: 1.19 mM<br>IC_{50}: 0.80 mM<br>IC_{50}: 1.28 mM	MTT	In vitro	Tamoxifen<br>IC_{50}: 0.012 mM<br>IC_{50}: 0.017 mM<br>IC_{50}: 0.006 mM	MDA-MB-231 (breast cancer cell line)<br>A-549 (lung carcinoma cell line)	[46]

		IC$_{50}$: 1.79 mM			IC$_{50}$: 0.030 mM	HT-29 (colon adenocarcinoma cell line)<br>MRC-5 (human fatal lung fibroblast)	
	Ostericum koreanum	ICG: 3.4% (at 25 μM after 24 h)<br>ICG: 43.5% (at 100 μM after 24 h)<br>ICG: 8.7% (at 25 μM after 48 h)<br>ICG: 76.2% (at 100 μM after 48 h)<br>ICG: 43.6% (at 25 μM after 72 h)<br>ICG: 93.3% (at 100 μM after 72 h)<br><br>CD: 19% (at 25 μM after 24 h)<br>CD: 22% (at 50 μM after 24 h)<br>CD: 28% (at 100 μM after 24 h)<br>CD: 18% (at 25 μM after 48 h)<br>CD: 25% (at 50 μM after 48 h)<br>CD: 35% (at 100 μM after 48 h)<br>CD: 15% (at 25 μM after 72 h)<br>CD: 32% (at 50 μM after 72 h)<br>CD: 50% (at 100 μM after 72 h)	trypan blue dye exclusion	In vitro	nd	human prostate carcinoma DU145 cell	[52]
	Ostericum koreanum	CC$_{50}$: 272.6 ± 6.9 μM	MTT	In vitro	Etoposide<br>CC$_{50}$: 46.9 ± 10.5 μM	Mice neuroblastoma neuro-2A cell	[51]
	Prangos uloptera	IC$_{50}$: 314 μg/mL	MTT	In vitro	nd	HeLa cell line	[63]
	Zanthoxylum flavum	IC$_{50}$: 8.9 μg/mL<br>No activity	XTT	In vitro	Doxorubicin<br>5-flurouracil	HL-60 (human leukemia cell line)<br>Vero cell line	[69]
	purchased	P_{app} (A→B): 0.47 ± 0.07 (× 10^{-6} cm/s) (at 3.82 μmol/L + VCR)<br>P_{app} (A→B): 0.50 ± 0.04 (× 10^{-6} cm/s) (at 19.08 μmol/L + VCR)<br>P_{app} (A→B): 0.52 ± 0.04 (× 10^{-6} cm/s) (at 76.30 μmol/L + VCR)	MTT	In vitro	VCR (at 264.46 μmol/L)<br>P_{app} (A→B): 0.37 ± 0.04 (× 10^{-6} cm/s)	MDCK-MDR1 cell monolayers	[106]
Enzyme inhibitory	*Angelica dahurica*	IC$_{50}$: 89.1 μM	Ellman (AChE inhibitory activity)	In vitro	Berberine<br>IC$_{50}$: 2.9 μM	-	[17]
	Angelica dahurica	IC$_{50}$: 69.3 ± 1.1 μg/mL	Ellman	In vitro	Eserine	-	[19]

			(AChE inhibitory activity)		IC_{50}: 0.51 ± 0.03 µg/mL		
	Angelica dahurica	IC_{50}: 359.2 ± 9.6 µM	BACE1 inhibitory activity	In vitro	LTEEISEVD(Statine)VAEF-OH<br>IC_{50}: 0.2 ± 0.01 µM	-	[22]
	Angelica koreana	IC_{50}: >20 µg/mL	testosterone 5α-reductase type I inhibitory	In vitro	Finasteride<br>IC_{50}: 19.8 µg/mL	LNCaP cell	[37]
	Angelica purpurascens	IC_{50}: 91.27 µg/mL<br>I: 19.36 ± 1.87% (against AChE)<br>I: 36.89 ± 1.23% (against BChE)<br>DS: -7.523 kcal/mol [against AChE (1EVE)]<br>DS: -4.232 kcal/mol [against BChE (1P0I)]<br>IC_{50}: 6.72 ± 0.98 µM (hCA I)<br>IC_{50}: 5.29 ± 0.98 µM (hCA II)	Anti-lipid peroxidation<br>Ellman (AChE inhibitory activity)<br>Molecular docking activity<br>Carbonic anhydrase isoenzymes I and II inhibition (cytosolic isoenzyme hCA I and II)	In vitro	-	-	[39]
	Levisticum officinale	DS: -7.764 kcal/mol [against 2-trans-enoyl-ACP reductase (InhA)]	Molecular docking activity		Isoniazid<br>DS: -6.013 kcal/mol	-	[60]
Insecticidal	*Ferulago trifida*	LC_{50}: 116.54 ppm	WHO	In vitro	na	*Anopheles stephensi*	[45]

		LC_{90}: 346.41 ppm	protocol				
Phytotoxic	*Petroselinum crispum*	GI: 70.21 ± 9.96% (of control)<br>TG: 93.33 ± 7.63% (of control)<br>RL: 26.41 ± 15.88% (of control)<br>SL: −24.37 ± 7.63% (of control)	Lettuce assay	In vitro	-	*Lactuca sativa* (0.06 g/L)	[56]
	Prangos uloptera	IC_{50}: 0.21 mg/mL (in seed germination)<br>IC_{50}: 0.59 mg/mL (in shoot growth)<br>IC_{50}: 0.62 mg/mL (in root growth)	Lettuce assay	In vitro	-	*Lactuca sativa*	[63]

AChE: acetylcholinesterase enzyme, AF-2: furylfuramide, AI: antifeedant index, BACE1: β-secretase, BChE: butyrylcholinesterase enzyme, BF: biofilm formation, BHT: butylated hydroxytoluene, C: concentration, CC_{50}: 50% cytotoxic concentration , CD: cell death, COD: colony diameter, DCFH-DA: dichloro-dihydro-fluorescein diacetate, DNP-HSA: dinitrophenyl-human serum albumin, DPPH: 2,2-diphenyl-1-picrylhydrazyl, DS: dock score, EC_{50}: half maximal effective concentration , ED_{50}: median effective dose, FRAP: Ferric reducing antioxidant power, FSE: ferrous sulphate equivalent, GI: germination index, HC: histamine content, hCA: human carbonic anhydrase, I: inhibition, IC_{50}: half maximal inhibitory concentration, ICG: inhibition of cell growth, I_{GABA}: GABA-induced chloride currents, IL-1β: interleukin, IZ: inhibition zone, LC_{50}: 50% lethal dose, LC_{90}: 90% lethal dose, L-NAME: L-N^G-Nitro arginine methyl ester, L-NMMA: N^G-monomethyl-L-arginine, L-NIO: *N*-(iminoethyl)-L-ornithine, LNCaP: androgen-sensitive human prostate adenocarcinoma, MBC: minimum bactericidal concentration, MDCK: Mardin Darby canine kidney cell, MelQ: 2-amino-3,4-di-methylimidazo[4,5-f]quinoline, MIC: minimum inhibitory concentration, MNNG: N-methyl-N′-nitro-N-nitrosoguanidine, MTT: 3-(4,5-Dimethylthiazol-2-yl)-2,5-diphenyltetrazolium bromide, na: not analysed, nd: not determined, NIH/3T3: mice embryonic fibroblast cell line, NO: nitric oxide, P_{app}: apparent permeability, PBTA-4: procarcinogen: 2-[2-(acetylamino)24-amino-5-methoxyphenyl]25-amino-7-bromo-4-chloro-2H-benzotriazole, PI: Pasteur institute, RAW 264.7: murine macrophage cell line, RBL-2H3: rat basophilic leukemia, RC_{50}: 50% reactive concentration, RL: root length, SL: shoot length, SRB: sulforhodamine B, TG: total germination, TNF-α: tumour necrosis factor, TV: tumour volume, TW: tumour weight, VCR: vincristine sulphate.
